# Supplementary material for: Growth monitoring and promotion program services utilization patterns between home-based and facility-based delivery methods: A comparative analysis
Source: PLoS One. 2025 Jun 5;20(6):e0324918. doi: 10.1371/journal.pone.0324918 (PMC12140421; doi:10.1371/journal.pone.0324918)
Supplement: S1 File — (DOCX) [file pone.0324918.s001.docx]

**Supporting file 1**

**S1 Table. Socio-demographic characteristics of sub-districts among the home-based and facility based GMP area**

| **Characteristics** | | **Home-based GMP** | | | **Facility-based GMP** | | | **p-value** |
| --- | --- | --- | --- | --- | --- | --- | --- | --- |
|  |  | **Sub-district 1** | **Sub-district 2** | **Sub-district 3** | **Sub-district 4** | **Sub-district 5** | **Sub-district 6** |  |
| Maternal age in years, mean (SD) | | 25.3 (5.7) | 26 (6.1) | 25.4 (5.9) | 25.8 (5.8) | 26.6 (5.9) | 25.7 (5.3) | <0.001 |
| Mothers’ education in years, mean (SD) | | 6.4 (3.5) | 6 (3.6) | 5.9 (3.5) | 6.1 (3.6) | 6.2 (3.7) | 6.1 (3.5) | 0.288 |
| Fathers’ education in years, mean (SD) | | 6.9 (3.3) | 6.8 (3.4) | 6.9 (3.4) | 7.4 (5.7) | 7.5 (3.4) | 7.1 (3.6) | 0.056 |
| Religion | |  |  |  |  |  |  |  |
|  | Muslim | 477 (98.4) | 546 (98.4) | 455 (95) | 559 (97.7) | 643 (98.3) | 292 (99.7) | <0.001 |
|  | Non-Muslim | 8 (1.7) | 9 (1.6) | 24 (5) | 13 (2.3) | 11 (1.7) | 1 (0.3) |  |
| Asset index | |  |  |  |  |  |  |  |
|  | Poor | 92 (19) | 226 (40.7) | 159 (33.2) | 215 (37.6) | 253 (38.7) | 80 (27.3) | <0.001 |
|  | Middle | 154 (31.8) | 183 (33) | 163 (34) | 214 (37.4) | 218 (33.3) | 88 (30) |  |
|  | Rich | 239 (49.3) | 146 (26.3) | 157 (32.8) | 143 (25) | 183 (28) | 125 (42.7) |  |
| Improved source of drinking water, n (%) | | 483 (99.6) | 554 (99.8) | 478 (99.8) | 572 (100) | 652 (99.7) | 290 (99) | 0.183 |
| Improved toilet facility, n (%) | | 455 (93.8) | 526 (94.8) | 454 (94.8) | 519 (90.7) | 609 (93.1) | 274 (93.5) | 0.076 |
| Proportion of boys, n (%) | | 243 (50.1) | 296 (53.3) | 250 (52.2) | 281 (49.1) | 332 (50.8) | 147 (50.2) | 0.771 |
| Child's age in Months, mean (SD) | | 9.9 (1.7) | 10 (1.8) | 9.6 (1.7) | 9.5 (1.8) | 9.9 (1.8) | 9.8 (1.8) | <0.001 |

**Details on statistical analysis**

Methods for assessing normality of continuous variables: We assessed the normality of continuous variables using histograms and quantile-normal (Q-Q) plots (qnorm). The continuous variables included the child’s length-for-age z-score, weight-for-age z-score, weight-for-length z-score, mother’s age (in years), mother’s and father’s years of completed formal education, and the child’s age (in months). To further examine deviations from a normal distribution, we conducted skewness and kurtosis tests for each variable.

Supplementary figure 1a: Histogram for Length-for-age Z-score

Supplementary figure 1b: Quantile Normal distribution plot for Length-for-age Z-score

Supplementary figure 2a: Histogram for weight-for-age Z-score

Supplementary figure 2b: Quantile Normal distribution plot for Weight-for-age Z-score

Supplementary figure 3a: Histogram weight-for-length Z-score

Supplementary figure 3b: Quantile Normal distribution plot for Weight-for-length Z-score

Supplementary figure 4a: Histogram of mother’s age in years

Supplementary figure 4b: Quantile Normal distribution plot for Mother’s age in years

Supplementary figure 5a: Histogram of the mother's level of education in years

Supplementary figure 5b: Quantile Normal distribution plot for the mother's level of education in years

Supplementary figure 6a: Histogram of the father's level of education in years

Supplementary figure 6b: Quantile Normal distribution plot for the father's level of education in years

Supplementary figure 7a: Histogram of child's age in months

Supplementary figure 7b: Quantile Normal distribution plot for child's age in month

**S2 Table: Skewness and kurtosis tests for continuous variables**

| **Indicators** | **n** | **Pr(Skewness)** | **Pr(Kurtosis)** | **Overall p-value** |
| --- | --- | --- | --- | --- |
| Child’s length-for-age Z-score | 3034 | 0.655 | 0.000 | 0.000 |
| Child’s weight-for-age Z-score | 3034 | 0.018 | 0.079 | 0.013 |
| Child’s weight-for-height Z-score | 3034 | 0.000 | 0.000 | 0.000 |
| Mother's age, years | 3035 | 0.000 | 0.049 | 0.000 |
| The mother’s level of education, years | 3038 | 0.000 | 0.437 | 0.001 |
| The father’s level of education, years | 2142 | 0.000 | 0.017 | 0.000 |
| Child's age, months | 3038 | 0.510 | 0.000 | 0.000 |

**Model with co-variates:**

**S3 Table: Predictors of GMP service utilization at home-based GMP program**

| **Characteristic^a^** | | **RR (95% CI)** | **p-value** | **ARR (95% CI)** | **p-value** |
| --- | --- | --- | --- | --- | --- |
| Caregivers heard about GMP or GMP card | | | | | |
|  | No | Reference |  | Reference |  |
|  | Yes | 42.4 (20.3, 88.6) | <0.001 | 37.4 (17.8, 78.5) | <0.001 |
| The caregiver is a member of an association/NGO/health program | | | | | |
|  | No | Reference |  | Reference |  |
|  | Yes | 2.6 (2.1, 3.2) | <0.001 | 1.3 (1.1, 1.5) | 0.001 |
| Caregivers' low interest in GMP service | | | | | |
|  | No | Reference |  | Reference |  |
|  | Yes | 0.5 (0.4, 0.7) | <0.001 | 0.7 (0.5, 0.9) | 0.001 |

RR: Risk ratio, CI: Confidence interval, ARR: Adjusted risk ratio, Growth monitoring and promotion, GMP; Non-governmental organization, NGO; and 1 = reference.

^a^The Model included caregivers who heard about GMP or GMP cards, were members of an NGO, and lacked interest in GMP service.
