# Supplementary material for: Growth monitoring and promotion program services utilization patterns between home-based and facility-based delivery methods: A comparative analysis
Source: PLoS One. 2025 Jun 5;20(6):e0324918. doi: 10.1371/journal.pone.0324918 (PMC12140421; doi:10.1371/journal.pone.0324918)
Supplement: S2 File — (DOCX) [file pone.0324918.s002.docx]

S2 File. Study questionnaire in English

| **Sl#** | **Questions** | | **Responses and codes** | | | |  |  |
| --- | --- | --- | --- | --- | --- | --- | --- | --- |
| 1. | Name of the subdistrict you (mother/caregiver) are currently living | | 1= Sub-district 1  2= Sub-district 2  3= Sub-district 3  4= Sub-district 4  5= Sub-district 5  6= Sub-district 6 | | | |  |  |
| 2. | How old are you (mother)? | | 10-50 (years) | | | |  |  |
| 3. | What is your current marital status (mother)? | | 01= unmarried  02 = married  03 = divorced/separated  04 = Widow | | | |  |  |
| 4. | Did you study in school (mother)? If yes, go to next question | | 01 = Yes  00 = No | | | |  |  |
| 5. | How many years of formal education do you have (mother)? | | 00-20 years | | | |  |  |
| 6. | What is your current occupation (mother)? | | __________________ | | | |  |  |
| 7. | How many years of formal education do your husband have? | | 00-20 years | | | |  |  |
| 8. | What is your religion? | | 01=Islam  02=Hindu  03= Buddhism  04= Christian  96= others (mention  --------------) | | | |  |  |
| 9. | How old is you child? | | _______ months ______days | | | |  |  |
| 10. | What is gender of your child | | 1= male  2= female | | | |  |  |
| 11 | What type of toilet do you use? (Ask the mother and observe) | | 11= flush or pour flush toilet  12= flush to piped sewer system  13= flush to septic tank  14= flush to pit latrine  15= flush to somewhere else  16= flush, don't know where  21= pit latrine ventilated  22= improved pit latrine  23= pit latrine with slab  24= pit latrine without slab/ open pit  31= composting toilet  41= bucket toilet  51= hanging toilet/hanging latrine  61= no facility/bush/field  96= others (mention  --------------) | | | |  |  |
| 12 | What do you usually do to make the water safer to drink? | | A= boil  B= add bleach/chlorine  C= strain through a cloth  D= use water filter (ceramic/ sand/composite/etc.)  E= Solar disinfection  F= Let it stand and settle  X= other (specify)  Z= don't know | | | |  |  |
| 13 | Does your household have (record all):  Electricity?  Solar electricity?  A radio?  A television?  A mobile telephone?  A non-mobile telephone?  A refrigerator?  An almirah/wardrobe?  An electric fan A DVD/VCD player?  A water pump?  An IPS/generator?  An air conditioner?  computer/laptop? | | 1= Yes  2= No | | | |  |  |
| 14 | Are you a member of an NGO program/project? | | 1= Yes  2= No | | | |  |  |
| 15 | Have you ever heard or seen GMP card? | | 1= Yes  2= No | | | |  |  |
| 16 | Do you know the purpose of the growth chart? | | 1= Yes  2= No | | | |  |  |
| 17 | Can you explain the colors in the GMP card growth chart? If yes, then go to the next question | | 1= Yes  2= No | | | |  |  |
| 18 | What does she understand by the color in the GMP card? | | | | | | | |
|  | Red | Orange/deep yellow | | Light yellow | Green | White | | |
|  | 99=Don’t know | 99=Don’t know | | 99=Don’t know | 99=Don’t know | 99=Don’t know | | |
|  | 1= severe malnutrition | 1= severe malnutrition | | 1= severe malnutrition | 1= severe malnutrition | 1= severe malnutrition | | |
|  | 2=Moderate malnutrition | 2=Moderate malnutrition | | 2=Moderate malnutrition | 2=Moderate malnutrition | 2=Moderate malnutrition | | |
|  | 3= Mild malnutrition | 3= Mild malnutrition | | 3= Mild malnutrition | 3= Mild malnutrition | 3= Mild malnutrition | | |
|  | 4= healthy state | 4= healthy state | | 4= healthy state | 4= healthy state | 4= healthy state | | |
|  | 5= overweight or height | 5= overweight or height | | 5= overweight or height | 5= overweight or height | 5= overweight or height | | |
|  | 8 = other (specify) | 8 = other (specify) | | 8 = other (specify) | 8 = other (specify) | 8 = other (specify) | | |
| 19 | What is your opinion about GMP card? | | 1= beneficial  2= non-beneficial/ no use  99 = don’t know | | | |  |  |
| 20 | Have you ever gone to a GMP service for your last child? If not, go to next question | | 1= Yes  2= No | | | |  |  |
| 21 | Why did you not go for a GMP service? (multiple response) | | 1. Long distance from home 2. Lack of transport/ poor road condition 3. No one to accompany 4. Busy in household chores 5. The community clinic was closed 6. Low quality of service 7. Religious reasons (the service is prohibited/should not be taken) 8. Service not given in my suitable time 9. Bad behavior of service providers 10. Lack of privacy 11. Long waiting time 12. Expensive service 13. Lack of medicine supply 14. Service does not benefit at all 15. No need for the service 16. Did not realize the importance of service 17. Family members did not consent 18. Did not know where to get the service   X. other (specify)   1. No benefits of getting the service 2. Did not feel the importance 3. Did not realize the importance of service | | | |  |  |
