# Supplementary material for: Growth monitoring and promotion program services utilization patterns between home-based and facility-based delivery methods: A comparative analysis
Source: PLoS One. 2025 Jun 5;20(6):e0324918. doi: 10.1371/journal.pone.0324918 (PMC12140421; doi:10.1371/journal.pone.0324918)
Supplement: S4 File — (DOCX) [file pone.0324918.s004.docx]

**Community Clinic Observation Checklist**

**Instructions:**

To strengthen the quality of growth monitoring, promotion, and other nutrition services, observers must follow a structured observation approach at community clinics throughout the study.

- **Date of Observation:** ___________________________
- **Location of Observation:** _________________________
- **Within Community Clinics under:**___________________________
- **Start Time:** __________________ **End Time:** __________________
- **Observer’s Name:** ___________________________________

**Observation Checklist:**

**1. Waiting Area:**

- **Seating arrangement:**
  00 = Inadequate
  01 = Adequate
- **Cleanliness:**
  00 = Not good
  01 = Good
  02 = Very good
  03 = Fair
- **Ventilation and lighting:**
  00 = Not good
  01 = Good
  02 = Very good
  03 = Fair
- **Toilet facilities:**
  00 = Not good
  01 = Good
  02 = Very good
  03 = Fair

**2. Approximate Waiting Time per Child: _____________ minutes**

**3. Did the service provider greet the mother/caregiver?**

01 = Yes
00 = No

**4. Supplies Available:**

- **Length board:**
  00 = Not available
  01 = Available and in good condition
  02 = Not in good condition
- **Weighing scale:**
  00 = Not available
  01 = Available and in good condition
  02 = Not in good condition
- **Growth chart/card:**
  00 = Not available
  01 = Available and in good condition
  02 = Not in good condition

**5. Was the child’s weight measured:**

00 = Incorrectly
01 = Correctly
02 = Fair

**6. Was the child’s length measured:**

00 = Incorrectly
01 = Correctly
02 = Fair

**7. How was the GMP information recorded?**

00 = Not recorded
01 = Correctly recorded on growth chart
02 = Recorded on chart but with errors
03 = Recorded in register

**8. Did the mother bring the growth monitoring card?**

00 = No
01 = Yes
02 = Lost
03 = Forgot to bring

**9. Did the provider explain the GMP findings to the mother?**

00 = No explanation
01 = Explained adequately
02 = Explained, but not adequately

**10. Did the provider ask about any other illness in the child?**

00 = No
01 = Yes

**11. Did the provider examine the child for other illness?**

00 = No
01 = Yes

**12. Did the provider give advice on feeding practices?**

00 = No advice
01 = Yes, adequate advice
02 = Yes, but inadequate advice

**13. Did the provider refer the child to the Upazila Health Complex?**

00 = No
01 = Yes

**14. Did the provider give a follow-up date?**

00 = No
01 = Yes

**15. Total time spent per child for GMP (length, weight, advice, growth chart):**

____________ minutes

**16. Who usually conducts GMP?**

00 = No one
01 = CHCP
02 = Health Assistant
03 = Family Planning Assistant
04 = NGO staff

**17. Who assists in GMP?**

00 = No one assists
01 = Health Assistant
02 = Family Planning Assistant
03 = NGO staff

**In-Depth Interview Guide with Government or NGO Service Providers**

**Instructions to Interviewer:**
Before the interview, inform the respondent and obtain written consent using the approved consent form.

**Main Interview Questions:**

1. Please explain your role in nutrition service delivery, especially for GMP (Growth Monitoring and Promotion) for children under 2 years of age.
   *(Probe: What are your specific tasks in GMP? What are the procedures? How is GMP implemented? How do you collaborate with partners? Who supervises you?)*
2. Have you received any training on GMP and child nutrition?
   *(Probe: Ask about training on GMP, IYCF, micronutrient supplementation, and community-based management of acute malnutrition—CMAM.)*
3. Can you explain the purpose of GMP for children under 2 years?
4. Please describe how you measure the weight and length of under-2 children at the community clinic.
5. Can you explain how to interpret the growth chart? How do you record GMP data?
   *(Probe: Try to understand how the growth chart is used during interviews.)*
6. Can you tell us about approved child feeding practices?
7. How do you provide nutrition counseling to mothers/caregivers?
   *(Probe: Do they use IYCF materials, flipcharts, posters, recipe demos, breastfeeding promotion, exclusive breastfeeding messaging, etc.?)
8. How do you keep track of children enrolled in GMP?
   *(Probe: What is the follow-up process in the community? How do community support groups engage in GMP?)*
9. Do you have printed SOPs or guidelines on child nutrition (e.g., GMP, micronutrient supplements, CMAM)?
10. Can you describe your experience of delivering GMP services?
11. Can you explain the barriers you face in providing child nutrition services?
12. What are your recommendations for improving GMP and child nutrition services at the primary care level?
    *(Probe: What are the key issues to address quickly, such as staffing, training, supplies, space, and community participation?)*

**Guidelines for interview with mothers/care givers of children less than 2 years of age**

**Instructions for the Interviewer:**
Before conducting the interview, please inform the respondent and obtain written consent using the approved consent form.

**Discussion with Mothers/Caregivers of Children Under 2 Years of Age**

1. Please describe in detail your experience of having your child’s weight and length measured at the community clinic.
   *(Instruction: Try to document experiences related to weight and length measurement at different stages, satisfaction with the procedures, waiting time, staff behavior, and the environment.)*
2. Has anyone ever explained to you about the measurement of your child’s weight and length? If yes, what did they say?
   *(Instruction: Refer to the growth chart and relate the respondent’s description accordingly.)*
3. What is your opinion about regular growth monitoring? Do you think it is important and necessary for your child’s health? If yes, please explain why it is important.
4. Does your child receive any medicine/micronutrient from the community clinic? If yes, what is it? What instructions were given by the community clinic staff regarding the intake of the medicine/micronutrient?
   *(Instruction: Request the mother to mention the name of the provider, if possible.)*
5. What could happen if your child’s weight falls below the expected weight? Please discuss.
6. What challenges do you face in receiving growth monitoring and other nutrition services from the community clinic? How do you overcome these challenges?
7. What is your advice for improving your child’s nutrition program?
