# Supplementary material for: Growth monitoring and promotion program services utilization patterns between home-based and facility-based delivery methods: A comparative analysis
Source: PLoS One. 2025 Jun 5;20(6):e0324918. doi: 10.1371/journal.pone.0324918 (PMC12140421; doi:10.1371/journal.pone.0324918)
Supplement: Attachment — Submitted filename: PONE-D-24-20319_reviewer (1).pdf [file pone.0324918.s007.pdf]

PLOS ONE

Caregiver utilization patterns in home-based and facility-based growth monitoring and promotion programs: A comparative analysis

--Manuscript Draft--

**Manuscript Number:**	PONE-D-24-20319
**Article Type:**	Research Article
**Full Title:**	Caregiver utilization patterns in home-based and facility-based growth monitoring and promotion programs: A comparative analysis
**Short Title:**	Caregivers Utilization to Home vs. Facility-Based Growth Monitoring Programs in Bangladesh
**Corresponding Author:**	Muttaquina Hossain, MBBS, MPH<br>ICDDRB: International Centre for Diarrhoeal Disease Research Bangladesh<br>Dhaka, BANGLADESH
**Keywords:**	Growth monitoring and promotion, Caregiver, Children, Service Utilization, Home, Facility, Bangladesh
**Abstract:**	Background: In Bangladesh, utilization of government health facility-based GMP services is notably low, prompting non-governmental organizations (NGOs) to collaborate with the government to enhance GMP service utilization through home-based delivery. Despite this, there is limited information available on home-based GMP service utilization. This study takes a unique approach by investigating the utilization of GMP services between home-based and facility-based programs and identifying key factors and barriers to caregiver engagement with GMP services in rural Bangladesh.<br>Methods: A comprehensive descriptive mixed-method study was conducted across six sub-districts over five months. Three sub-districts with home-based GMP services provided by NGOs were compared with three neighboring sub-districts offering government facility-based GMP services. A total of 3038 mothers and children under one-year-old were included. GMP utilization was defined as caregivers receiving GMP services at least once in the child's lifetime. Quantitative surveys and qualitative approaches were used for data collection. Multiple logistic regression was employed to determine statistically significant associations between GMP utilization and independent variables at p-value <0.05 and adjusted risk ratio (ARR) values with a 95% confidence interval (CI). Thematic analysis of qualitative data was performed. Results were triangulated across sources.<br>Results: Home-based GMP services exhibited higher utilization rates, with more children receiving weight and length measurements and caregivers receiving counseling than facility-based services (40% vs. 0% utilization, respectively). Caregivers' utilization of GMP services in home-based areas was positively influenced by their knowledge of GMP or GMP cards (Adjusted risk ratio, ARR: 37.4, 95% confidence interval, 95% CI: 17.8,78.5) and their involvement with an NGO, association, or health program (ARR: 1.3, 95% CI: 1.1, 1.5). Lack of caregiver interest and awareness of GMP services emerged as significant barriers.<br>Conclusion: GMP service utilization remains low in some parts of rural Bangladesh. Tailored healthcare solutions addressing caregivers' needs are crucial to improving GMP program coverage and utilization rates.
**Order of Authors:**	Muttaquina Hossain, MBBS, MPH
	Md. Ahshanul Haque, Masters in Statistics
	A.S.G Faruque, MBBS
	Tahmeed Ahmed, PhD
	Ulla Ashorn, PhD
	Per Ashorn, PhD
**Additional Information:**	
**Question**	**Response**

Powered by Editorial Manager® and ProduXion Manager® from Aries Systems Corporation

**Financial Disclosure**<br><br>Enter a financial disclosure statement that describes the sources of funding for the work included in this submission. Review the submission guidelines for detailed requirements. View published research articles from *PLOS ONE* for specific examples.<br><br>This statement is required for submission and **will appear in the published article** if the submission is accepted. Please make sure it is accurate.<br><br>**Funded studies**<br>Enter a statement with the following details:<br>• Initials of the authors who received each award<br>• Grant numbers awarded to each author<br>• The full name of each funder<br>• URL of each funder website<br>• Did the sponsors or funders play any role in the study design, data collection and analysis, decision to publish, or preparation of the manuscript?<br><br>Did you receive funding for this work?	The author(s) received no specific funding for this work.
**Competing Interests**<br><br>Use the instructions below to enter a competing interest statement for this submission. On behalf of all authors, disclose any competing interests that could be perceived to bias this work—acknowledging all financial support and any other relevant financial or non-financial competing interests.<br><br>This statement is required for submission and **will appear in the published article** if the submission is accepted. Please make sure it is accurate and that any funding sources listed in your Funding Information later in the submission form are also declared in your Financial Disclosure statement.	The authors have declared that no competing interests exist.

View published research articles from *PLOS ONE* for specific examples.<br><br>**NO authors have competing interests**<br><br>Enter: *The authors have declared that no competing interests exist.*<br><br>**Authors with competing interests**<br><br>Enter competing interest details beginning with this statement:<br><br>*I have read the journal's policy and the authors of this manuscript have the following competing interests: [insert competing interests here]*<br><br>* typeset	
**Ethics Statement**<br><br>Enter an ethics statement for this submission. This statement is required if the study involved:<br><br>• Human participants<br>• Human specimens or tissue<br>• Vertebrate animals or cephalopods<br>• Vertebrate embryos or tissues<br>• Field research<br><br>Write "N/A" if the submission does not require an ethics statement.<br><br>General guidance is provided below. Consult the submission guidelines for detailed instructions. **Make sure that all information entered here is included in the Methods section of the manuscript.**	The Institutional Review Board (IRB) of icddr,b approved the study (PR-17123, version 1.02, dated 6th March 2018). Written informed consent with full disclosure about the study was taken from the participants before all interviews. The privacy of the respondents, their anonymity, and the confidentiality of data/information identifying the study participants were strictly maintained. Only the principal investigators of this study had access to information on personal identification and other sensitive information that the respondents provided.

Powered by Editorial Manager® and ProduXion Manager® from Aries Systems Corporation

**Format for specific study types**<br><br>**Human Subject Research (involving human participants and/or tissue)**<br>• Give the name of the institutional review board or ethics committee that approved the study<br>• Include the approval number and/or a statement indicating approval of this research<br>• Indicate the form of consent obtained (written/oral) or the reason that consent was not obtained (e.g. the data were analyzed anonymously)<br><br>**Animal Research (involving vertebrate animals, embryos or tissues)**<br>• Provide the name of the Institutional Animal Care and Use Committee (IACUC) or other relevant ethics board that reviewed the study protocol, and indicate whether they approved this research or granted a formal waiver of ethical approval<br>• Include an approval number if one was obtained<br>• If the study involved *non-human primates*, add *additional details* about animal welfare and steps taken to ameliorate suffering<br>• If anesthesia, euthanasia, or any kind of animal sacrifice is part of the study, include briefly which substances and/or methods were applied<br><br>**Field Research**<br><br>Include the following details if this study involves the collection of plant, animal, or other materials from a natural setting:<br>• Field permit number<br>• Name of the institution or relevant body that granted permission	
**Data Availability**<br><br>Authors are required to make all data underlying the findings described fully available, without restriction, and from the time of publication. PLOS allows rare exceptions to address legal and ethical concerns. See the PLOS Data Policy and FAQ for detailed information.	No - some restrictions will apply

Powered by Editorial Manager® and ProduXion Manager® from Aries Systems Corporation

A Data Availability Statement describing where the data can be found is required at submission. Your answers to this question constitute the Data Availability Statement and **will be published in the article**, if accepted.<br><br>**Important:** Stating 'data available on request from the author' is not sufficient. If your data are only available upon request, select 'No' for the first question and explain your exceptional situation in the text box.<br><br>Do the authors confirm that all data underlying the findings described in their manuscript are fully available without restriction?	
**Describe where the data may be found in full sentences. If you are copying our sample text, replace any instances of XXX with the appropriate details.**<br><br>• If the data are **held or will be held in a public repository**, include URLs, accession numbers or DOIs. If this information will only be available after acceptance, indicate this by ticking the box below. For example: *All XXX files are available from the XXX database (accession number(s) XXX, XXX.*).<br>• If the data are all contained **within the manuscript and/or Supporting Information files**, enter the following: *All relevant data are within the manuscript and its Supporting Information files.*<br>• If neither of these applies but you are able to provide **details of access elsewhere**, with or without limitations, please do so. For example:<br><br>*Data cannot be shared publicly because of [XXX]. Data are available from the XXX Institutional Data Access / Ethics Committee (contact via XXX) for researchers who meet the criteria for access to confidential data.*<br><br>*The data underlying the results presented in the study are available from (include the name of the third party*	Data cannot be shared publicly because of institutional data protection policy. Data are available from the icddr,b Institutional Data Access / Ethics Committee (contact via +8801711428989) for researchers who meet the criteria for access to confidential data.

and contact information or URL).<br>• This text is appropriate if the data are owned by a third party and authors do not have permission to share the data.<br><br>* typeset	
Additional data availability information:	

Powered by Editorial Manager® and ProduXion Manager® from Aries Systems Corporation

Manuscript
Click here to access/download;Manuscript;GMP manuscript_home vs. facility_22 May 2024.docx

Title: Caregiver utilization patterns in home-based and facility-based growth monitoring and promotion programs: A comparative analysis

Author: Muttaquina Hossain[1*], Md. Ahshanul Haque[1], A.S.G. Faruque[1], Tahmeed Ahmed[1, 2], Ulla Ashorn[3], Per Ashorn[3,4]

Affiliations:

[1] Nutrition Research Division, icddr,b, Mohakhali, Dhaka, Bangladesh

[2] James P Grant School of Public Health, BRAC University, Mohakhali, Dhaka, Bangladesh

[3] Center for Child, Adolescent and Maternal Health Research, Faculty of Medicine and Health Technology, Tampere University, Tampere, Finland

[4] Department of Paediatrics, Tampere University Hospital, Tampere, Finland

Corresponding author: E-mail: muttaquina@icddrb.org; muttaquina@gmail.com (MH)

Abstract

Background: In Bangladesh, utilization of government health facility-based GMP services is notably low, prompting non-governmental organizations (NGOs) to collaborate with the government to enhance GMP service utilization through home-based delivery. Despite this, there is limited information available on home-based GMP service utilization. This study takes a unique approach by investigating the utilization of GMP services between home-based and facility-based programs and identifying key factors and barriers to caregiver engagement with GMP services in rural Bangladesh.

Methods: A comprehensive descriptive mixed-method study was conducted across six sub-districts over five months. Three sub-districts with home-based GMP services provided by NGOs were compared with three neighboring sub-districts offering government facility-based GMP services. A total of 3038 mothers and children under one year old were included. GMP utilization was defined as caregivers receiving GMP services at least once in the child's lifetime. Quantitative surveys and qualitative approaches were used for data collection. Multiple logistic regression was employed to determine statistically significant associations between GMP utilization and independent variables at p-value <0.05 and adjusted risk ratio (ARR) values with a 95% confidence interval (CI). Thematic analysis of qualitative data was performed. Results were triangulated across sources.

Results: Home-based GMP services exhibited higher utilization rates, with more children receiving weight and length measurements and caregivers receiving counseling than facility-based services (40% vs. 0% utilization, respectively). Caregivers' utilization of GMP services in home-based areas was positively influenced by their knowledge of GMP or GMP cards (Adjusted risk ratio, ARR: 37.4, 95% confidence interval, 95% CI: 17.8,78.5) and their

involvement with an NGO, association, or health program (ARR: 1.3, 95% CI: 1.1, 1.5). Lack of caregiver interest and awareness of GMP services emerged as significant barriers.

Conclusion: GMP service utilization remains low in some parts of rural Bangladesh. Tailored healthcare solutions addressing caregivers' needs are crucial to improving GMP program coverage and utilization rates.

Key Words: Growth monitoring and promotion, Children, Service Utilization, Program, Bangladesh

Introduction

Growth Monitoring and Promotion (GMP) represent integral components of preventive and promotional endeavors, employing recurrent measurements and analyses of child growth to discern growth faltering (1). This approach aims to foster communication and interaction with caregivers, promoting optimal child development (1). The introduction of the promotion component by UNICEF in the mid-1980s augmented the concept, rendering GMP a vital facet of nutrition programs beneficial to effective malnutrition management in children within low- and middle-income country (LMIC) settings (2).

The effectiveness of GMP programs depends on factors like program coverage, caregiver interactions with healthcare providers, health worker performance, facility readiness, community engagement, appropriateness of delivery platform, and sociocultural contexts, stressing the need for optimal resource mobilization (1, 3). Primarily implemented through health facilities in LMICs, research on GMP programs has predominantly focused on identifying deficiencies in facility-level readiness and supply-side inadequacies (1, 4-6). These investigations often neglect the crucial assessment of acceptability and feasibility from the perspectives of caregivers and communities, along with their sociocultural contexts. The effectiveness of facility-based GMP programs in LMICs in improving caregiver utilization and reducing child undernutrition remains uncertain (5, 7-9). To bridge this gap in facility-based GMP service delivery, certain LMICs in Africa and Asia have adopted community and home-based platforms for GMP service delivery (1). The effectiveness of these decentralized approaches hinges upon factors such as the communication and counseling skills of health workers, the motivation and willingness of caregivers to engage with the service, and the accessibility of appropriate referrals and interventions (10). However, despite the promise of these alternatives, most home/community-

based GMP services remain isolated and lack linkage with the broader health system, posing challenges to sustainability and scalability (1). Consequently, the outcomes of home-based GMP services exhibit variability across diverse contexts and regions (11).

In the ongoing debate surrounding the most effective delivery platform for GMP services in Low- and Middle-Income Countries (LMICs) with limited resources. This study aims to delve into caregiver GMP service utilization across both home-based and facility-based settings. The primary objectives are twofold: firstly, to ascertain whether disparities exist in GMP service utilization between home-based and facility-based programs, and secondly, to pinpoint and analyze the principal barriers hindering caregiver engagement with GMP services in these respective contexts. By comprehensively examining the dynamics of service utilization and the multifaceted factors influencing caregiver participation, valuable information will be provided to researchers, program developers, public health practitioners, and policymakers. Through a rigorous analysis of these anticipated outcomes, evidence-based decision-making processes will be fostered, ultimately enhancing the utilization of GMP services for caregivers and improving healthcare outcomes in LMICs.

Materials and methods

Study design, period, and site

A community-based cross-sectional study employing a mixed-method approach was conducted in six rural sub-districts of Bangladesh's Mymensingh district from August to December 2019. In this area, the Government of Bangladesh administered Growth Monitoring and Promotion (GMP) services via health facility-based platforms, notably community clinics (CC). These CCs, which are community-based government healthcare facilities, cater to the health, family planning, and nutrition needs of 6,000 or more households (12). This study is a sub-study of a larger study that aimed to evaluate the effectiveness of GMP programs in improving child nutritional status (13).

To understand the home-based and facility-based GMP service delivery models, three sub-districts were selected where a non-governmental organization (NGO) supplemented the government's facility-based GMP services with home-based initiatives. Concurrently, three neighboring sub-districts were chosen where only the government's facility-based GMP services were available, without any NGO involvement. We compared caregivers' knowledge of GMP service, levels of GMP service utilization, and barriers to GMP service utilization in these two settings. This approach allowed for a comparative analysis of GMP service delivery in varied contexts within the Mymensingh district.

Selection and Recruitment of Study Participants

The study included children aged 6-23 months and their mothers/caregivers in Community clinics' catchment areas. Eligibility criteria encompassed children free from acute/chronic illnesses, severe stunting, wasting, or underweight, not enrolled in similar

programs, and whose caregivers provided consent. The youngest child in each household was designated as the index child, with twins excluded to prevent potential biases. In case of participant absence, the next eligible household was approached during data collection.

Components of growth monitoring and promotion (GMP)

The GMP program was designed to include regular check-ups for child growth, happening every three months at health facilities. The components of GMP are intended, not documented, actions. Health workers were advised to counsel mothers and caregivers on their children's nutrition, measure the weight, length, or mid-upper arm circumference (MUAC) of all under-5-year-old children, and plot the results on a Growth Monitoring (GM) chart in the GMP card. If a child was acutely ill or not growing well, medical treatment or referrals were indicated (14).

Facility-based GMP service delivery

The Government of Bangladesh (GoB) has implemented a Growth Monitoring and Promotion (GMP) program in its Community Clinics (CCs) since 2011. Each CC in the current study was meant to have three healthcare staff responsible for providing GMP to every child under five years of age, whether they visited for illness or routine check-ups. The GoB had developed a National Guideline for GMP implementation. It was responsible for training the CC staff and providing the necessary equipment and supplies. Despite consistency in GMP components as described above (14), there has been a concern about the need for more active supervision at the CCs (15).

Home-based GMP service delivery

During the study period in Mymensingh, a single NGO partnered with the government to support the GMP program in certain sub-districts. The NGO provided extra support by engaging its staff for home-based GMP services. They offered GMP sessions at home for children using a tracking system. The NGO aimed to improve GMP utilization by creating counseling tools and ensuring a steady supply of GMP cards to caregivers at home. The GMP components were aligned with the previously outlined methodology (16).

GMP Service Utilization

Caregivers' GMP service utilization was defined as whether they received GMP services at home or went to the facility for the service even once during the child's lifetime during the survey.

Data processing and analysis

A. Quantitative data

Fourteen data collectors conducted a quantitative survey with mothers/caregivers of children aged 6-12 months in their households, using a pretested semi-structured questionnaire. The survey questionnaire covered topics including household socio-demographics, water sanitation and hygiene practices, knowledge and perception of GMP services, utilization of GMP services, perceived barriers in GMP service utilization, and child anthropometry.

Data collectors directly observed households' drinking water sources and toilets. Improved toilets were defined as flush toilets, ventilated improved pit latrines, traditional pit latrines with a slab, or composting toilets; improved drinking water sources were classified as water from tubewells (17). The data collectors utilized Bangladesh government-approved GMP

cards and asked caregivers about their knowledge of the cards, showing growth charts for interpretation. Caregivers' responses were recorded in closed-ended questionnaires. If caregivers had not utilized GMP services, they were asked for reasons via a multiple-option choice questionnaire.

Child anthropometry followed WHO standard operating procedures (18), with staff initially trained by certified gold standards at icddr,b. Measurements were taken using a Seca Infantometer for length and a Seca 727 Baby Scale for weight, ensuring daily precision through calibration and repeated readings when necessary. All measuring tools were placed on flat surfaces, and data collectors noted the readings when participants became steady on the scale. One data collector read the measurement, and another recorded it on the form.

The completed questionnaires were securely transported to icddr,b and received by data management assistants and a statistician for entry and analysis using MS Access and STATA software version 15, respectively. The study statistician and investigators decoded caregivers' responses on GMP growth card questionnaires and determined "correct" responses, e.g., if a caregiver mentioned that the "red color zone" in the growth chart means the child is severely malnourished, it was considered "correct" by the investigators. The study team assessed caregivers' responses to GMP growth card questionnaires for accuracy. Child growth parameters were calculated based on WHO standards (19), and a wealth index was derived from household assets (20).

Data were checked for normality using Skewness/Kurtosis tests, and outliers were managed according to standard statistical/data analysis procedures. Categorical variables were summarized using proportions and frequencies, while normally distributed continuous variables were summarized using means and standard deviations. Student's t-tests compared counts with a

normal distribution, and the chi-square distribution test compared qualitative variables for both home-based and facility-based GMP arms. The differences in caregivers' GMP knowledge between home-based and facility-based GMP proportions were reported using risk ratios (RR) with 95% confidence intervals (95% CI) without adjustments. Simple logistic regression models were used to determine caregivers' level of GMP service utilization and barriers to GMP service utilization.

Predictors of caregivers' GMP utilization were explored using logistic regression models only for the home-based group due to the absence of GMP service utilization among facility-based caregivers. The study examined factors influencing caregiver GMP service utilization versus non-utilization. All independent variables significantly associated with GMP utilization (dependent variable and primary outcome) in bivariate models and biologically plausible variables were included in the final multivariable models. The multivariable models were adjusted for relevant covariates: caregiver awareness of GMP or GMP card, NGO membership, and caregiver's lack of demand for GMP service. Estimated associations were described as adjusted risk ratios (ARR) with 95% confidence intervals (CIs). Results with p-values <0.05 were considered statistically significant.

B. Qualitative data collection

Qualitative approaches were applied to enhance the quantitative survey findings. The study employed one moderator and one interviewer proficient in conducting qualitative interviews and analysis. The qualitative interviews were carried out by trained moderators and interviewers whose native language was Bangla. Interviews were conducted in Bangla and later translated into English. Open-ended questionnaires and guidelines were used for qualitative interviews, along with checklists for Community Clinic (CC) observations. These tools included

questions on caregivers' GMP knowledge, perception, and practices, as well as CC-related GMP service information.

Two qualitative researchers (IJ and ZI) employed a thematic analysis for all qualitative information. Qualitative analysis included thematic descriptions, analysis, and quotations from respondents. Transcribed interviews were coded based on predetermined themes (a priori), subthemes, and emergent issues. Trustworthiness was ensured through four principles: credibility, transferability, conformability, and dependability (21). Intercoder or synchronic reliability was utilized to gauge the agreement between independent coders of the data. An agreement was assessed during the analysis when the researchers independently coded the same interviews. Triangulation was conducted between methods and participants, validating information by triangulating data from different approaches.

Sample Size and Sampling Technique

All the data of the participants enrolled in the larger study (3038 mother-child pairs) were included in the quantitative analysis (13). Only some relevant participants were interviewed for the qualitative part. The number of qualitative interviews was decided following an iterative process of achieving information saturation.

Ethical considerations

The Institutional Review Board (IRB) of icddr,b approved the study (PR-17123, version 1.02, dated 06 March 2018). Written informed consent with full disclosure about the study was taken from the participants before all interviews. The privacy of the respondents, their anonymity, and the confidentiality of data/information identifying the study participants were strictly maintained. Only the principal investigators of this study had access to information on personal identification and other sensitive information that the respondents provided.

Results

Sociodemographic characteristics of the study participants

A total of 3038 mother-child dyads were enrolled in this study between August and December 2019, evenly split between home-based and facility-based service areas (Figure 1). Sixteen qualitative in-depth interviews (IDIs) were conducted with CHCPs and 20 with mothers of enrolled children. In addition, daylong indirect observations were conducted at 23 CCs.

Children's average age was 9.8 months, with a 1:1 male-to-female ratio in both groups (home-based vs. facility-based: 51.9% vs. 50.0%). Children's nutritional status (LAZ, WAZ, and WHZ) was comparable among the groups. Caregivers' age, sex, and education level distributions were similar between both groups (Table 1). Approximately 98% of household members in both areas identified as Muslim, with 94% and 92% having improved toilets in home-based and facility-based GMP service areas, respectively (Table 1). 44% of caregivers in home-based GMP areas and 38% in facility-based GMP areas were from poor families (Table 1).

Table 1. Caregiver's sociodemographic characteristics

Characteristic	Home-based GMP<br>N = 1,519	Facility-based GMP<br>N = 1,519
Child age in months, mean (SD)	9.7 (1.8)	9.9 (1.7)
Child's weight-for-age Z-score (WAZ), mean (SD)	-1.1 (1.1)	-1.1 (1.1)
Child's length-for-age Z-score (LAZ), mean (SD)	-1.3 (1.1)	-1.4 (1.1)
Child's weight-for-length Z-score (WHZ), mean (SD)	-0.48 (0.03)	-0.40 (0.03)
Proportion of boys, n (%)	760 (50.0)	789 (51.9)
Mother's age in years, mean (SD)	26.1 (5.8)	25.6 (5.9)
Mother's education in years, mean (SD)	6.1 (3.6)	6.1 (3.6)
Mother's occupation, n (%)		
Housewife/homemakers	1,474 (97.0)	1,487 (97.9)
Fathers' education in years, mean (SD)	7.4 (4.5)	6.9 (3.4)
Religion, n (%)		
Islam	1,494 (98.4)	1,478 (97.3)
Hinduism/Buddhism/Christian	25 (1.6)	41 (2.7)
Improved toilet facility, n (%)	1,402 (92.3)	1,435 (94.5)
Improved source of drinking water, n (%)	1,514 (99.7)	1,515 (99.7)
Wealth index, n (%)		
Poor	667 (44.0)	577 (38.0)
Middle	430 (28.3)	461 (30.3)
Rich	420 (27.7)	481 (31.7)

Caregiver's knowledge and perception of GMP service

Caregivers from home-based GMP areas had higher GMP knowledge levels compared to those in facility-based areas. In home-based GMP areas, a higher proportion of caregivers (67%) had heard about GMP or GMP cards compared to facility-based GMP areas (0.4%) (RR: 169, 95% CI: 76, 376; respectively) (Table 2). However, only 17% of caregivers in home-based GMP areas correctly understood the purpose of the growth chart and explained the meaning of different colors compared to less than 1% in facility-based GMP areas (RR: 127.5 and 122.5, 95% CI: 32, 512, and 31, 492, respectively). More than half of the caregivers (51%) in home-based GMP service areas perceived GMP service as beneficial, while none from facility-based regions did.

Table 2: Caregiver's knowledge of GMP service in facility and home-based GMP program

**Indicators**	**Home-based GMP** N = 1,519	**Facility-based GMP** N = 1,519	**RR (95% CI)**	**p-value**
Caregivers heard about GMP or GMP card	1,014 (66.8)	6 (0.4)	169 (76, 375.9)	<0.001
Caregivers know the purpose of the growth chart	255 (16.8)	2 (0.1)	127.5 (31.8, 511.6)	<0.001
Caregivers can correctly explain the colors in the GMP card growth chart	245 (16.1)	02 (0.13)	122.5 (30.5, 491.6)	<0.001
Caregivers mentioned GMP service as "beneficial."	777 (51.2)	0 (0)	-	-

Growth monitoring and promotion, GMP; confidence interval, CI; Risk Ratio, RR.

Caregiver's level of GMP service utilization

GMP service utilization was significantly higher in home-based areas compared to facility-based areas. In home-based GMP service areas, 61% of caregivers had received GMP cards (Table 3), while none had done so in facility-based GMP areas. Significantly more children underwent weight measurement, length measurement, and their caregivers received growth monitoring-specific counseling in home-based GMP service compared to facility-based GMP service (81% vs. 21%; 3.4% vs. 0.6%; and 55% vs. 06%, RR: 4, 5.7 and 9.5, respectively). However, only 30 out of 1519 children and their caregivers received all three major components of GMP services (child weight, length measurement, and GM-specific counseling) in a single GMP visit at home-based GMP services (Table 3). Children and their caregivers in facility-based areas never received all three components altogether. Overall, 40% of caregivers from home-based GMP service areas utilized GMP service compared to 0% in facility-based GMP service (Table 3).

Table 3. Levels of GMP service utilization in facility and home-based GMP program

**Indicators**	**Home-based GMP**, N = 1,519	**Facility-based GMP**, N = 1,519	**RR (95% CI)**	**p-value**
Caregivers received a GMP card	926 (60.9)	0 (0)	-	-
The child weighed at least once	1231 (81.0)	311 (20.5)	4.0 (3.6, 4.4)	<0.001
The child length was measured at least once	51 (3.36)	09 (0.59)	5.7 (2.8, 11.5)	<0.001
Caregivers got child GM-specific counseling	837 (55.2)	88 (5.4)	9.5 (7.7, 11.7)	<0.001
The child received all major components of GMP (length, weight, and GM-specific counseling) altogether	30 (1.97)	0 (0)	-	-
Caregivers utilized GMP services	603 (39.7)	0 (0)	-	-

Growth monitoring, GM; Growth monitoring and promotion, GMP; confidence interval, CI; Risk Ratio, RR.

Barriers to caregivers' access and utilization of GMP services

Four major barriers were identified as reasons for caregivers' underutilization of GMP services. Among these, caregivers' lack of knowledge about the availability of GMP services at the nearest health facility was a significant barrier in both areas. Caregivers from facility-based GMP areas had significantly less knowledge about GMP service availability compared to those in home-based service areas (52% vs. 61%, respectively; RR: 0.9, 95% CI: 0.8, 0.9). Additionally, caregivers' demand for GMP service was significantly lower in facility-based areas compared to home-based GMP areas (55% vs. 44%, respectively; RR: 0.7, 95% CI: 0.6, 0.9). More caregivers from home-based GMP service areas reported closure of health facilities/absence of service providers (1%) and being preoccupied with household work (4%) as significant barriers to accessing and utilizing GMP services, compared to facility-based GMP service areas (0.2% and 0.3%, respectively).

In-depth interviews (IDIs) with mothers/caregivers provided insight into the obstacles to accessing GMP services in both settings. Mothers/caregivers in the home-based GMP area mainly relied on NGO staff for GMP service delivery at home, bypassing visits to CCs for GMP services. In contrast, mothers in facility-based GMP areas reported that government community health workers never visited their communities or homes to inform them about GMP services at CCs or the nearest health facilities. Caregivers from both areas expressed unawareness of GMP services available at CCs or nearby health facilities. Additionally, caregivers mentioned a preference for accessing health services/programs that offered incentives or immediate benefits, such as vaccines, cash, food, vitamins, or supplements. As neither the home-based nor the facility-based GMP program provided such incentives, mothers/caregivers lacked interest in utilizing these services.

Table 4: Barriers to GMP service utilization in the facility and home-based GMP program

**Indicators**	**Home-based GMP** N = 1,519	**Facility-based GMP** N = 1,519	**RR (95% CI)**	**p-value**
The caregiver was preoccupied with household chores and did not go for the GMP service	56 (3.7)	5 (0.3)	11.2 (4.5, 27.9)	<0.001
The health facility was closed/no healthcare provider at the facility	14 (0.9)	3 (0.2)	4.6 (1.3, 16.2)	0.015
Caregivers' lack of interest in GMP services at an individual level	668 (44.0)	834 (54.9)	0.7 (0.6, 0.9)	0.006
Lack of knowledge of GMP service availability at the nearest health facility	798 (52.5)	919 (60.5)	0.9 (0.8, 0.9)	<0.001

Multiple responses.

Growth monitoring and promotion, GMP; confidence interval, CI; Risk Ratio, RR.

Predictors of caregivers' GMP service utilization

Utilization of GMP services was positively associated with caregivers' knowledge of GMP or GMP card (Adjusted risk ratio, ARR: 37.4, 95% confidence interval, 95% CI: 17.8,78.5) and membership of an NGO/association/health program (ARR: 1.3, 95% CI: 1.1, 1.5) (Table 5). A key barrier to GMP service utilization was caregivers' low interest in the service (ARR: 0.7; 95% CI: 0.5, 0.9).

Table 5. Predictors of GMP service utilization at home-based GMP program

Characteristic	RR (95% CI)	p-value	ARR (95% CI)	p-value
Caregivers heard about GMP or GMP card				
No	Reference		Reference	
Yes	42.4 (20.3, 88.6)	<0.001	37.4 (17.8, 78.5)	<0.001
The caregiver is a member of an association/NGO/health program				
No	Reference		Reference	
Yes	2.6 (2.1, 3.2)	<0.001	1.3 (1.1, 1.5)	0.001
Caregivers' low interest for GMP service				
No	Reference		Reference	
Yes	0.5 (0.4, 0.7)	<0.001	0.7 (0.5, 0.9)	0.001

RR: Risk ratio, CI: Confidence interval, ARR: Adjusted risk ratio, Growth monitoring and promotion, GMP; Non-governmental organization, NGO; and 1 = reference.

The Model was adjusted for caregivers who heard about GMP or GMP cards, were members of an NGO, and lacked demand for GMP service.

Discussion

The study aimed to assess caregivers' knowledge, perception, utilization, and barriers to accessing Growth Monitoring and Promotion (GMP) services in rural Bangladesh, comparing home-based and facility-based settings. In a sample of 1519 families from 3 rural subdistricts, caregivers from home-based GMP areas had higher GMP knowledge levels compared to facility-based GMP areas. More caregivers from home-based GMP service areas received GMP cards than from facility-based areas. Sixty-one percent of caregivers received GMP cards at home-based GMP, whereas none did in facility-based regions due to a lack of supply. More children were measured for weight and length at home, and their caregivers received growth monitoring-specific counseling compared to facilities. GMP service utilization was higher among caregivers at home compared to facility-based GMP services (40% vs. 0%, respectively).

The validity and reliability of the study findings could theoretically have been reduced by several factors, including the sole presence of one NGO offering home-based GMP services, the longstanding integration of this NGO within the community affecting caregivers' healthcare choices, the accessibility of home-based GMP services, and the relatively brief duration of the study. Nonetheless, the presence of similar sociodemographic characteristics among participants helped alleviate potential biases arising from the NGO's sole presence. Despite the potential impact of the NGO and the accessibility of home-based GMP services, the low utilization rates in the home-based GMP area indicate a limited effect. However, despite the study's short duration, its findings offer valuable insights into GMP utilization trends in rural settings. These findings underscore that caregivers of infants in rural Bangladesh significantly engaged with home-based GMP services, while facility-based services were not used at all. The validity and

reliability of these study findings are upheld through rigorous observation, robust study design, and mitigation of potential biases.

Our finding on low service utilization despite better knowledge of GMP is consistent with other LMIC settings (6, 22, 23). No other study has ever documented no GMP use in facilities, which is unique to this setting and contrasts with the existing LMIC GMP program findings (5, 22, 23, 26). This contrasting finding may be due to the influence of differences in cultural beliefs, social norms, and contextual factors (24). Caregivers from both home- and facility-based GMP settings prefer curative services over preventive services like GMP, which is also expected in LMIC settings (6, 25, 26). They only take their child to health facilities when sick, either for treatment or to obtain free medicines (27). Additionally, caregivers prefer incentives or immediate benefits, such as vaccines or cash, highlighting the importance of addressing these preferences to increase the utilization of GMP services, which several other studies also reported (5-7, 25, 26).

Caregivers' knowledge, perceptions, and barriers differ between home-based and facility-based GMP areas. Home-based services are better utilized due to NGO presence (11, 28, 29). However, gaps exist in understanding growth charts, requiring clearer explanations from health workers (5, 30). Reliance on NGOs in home-based areas and limited government visits in facilities hinder caregivers' GMP service awareness. The lack of GMP card supply in facilities contributes to non-utilization (26, 31, 32). Barriers like health facility closures and household responsibilities explain low utilization rates (6).

The findings emphasize the necessity for GMP programs tailored to caregivers' needs (4), highlighting differences between home and facility areas. Clear communication strategies are needed to address gaps in understanding growth charts (30). Improvement is required to ensure

caregivers receive all GMP components during home visits. Barriers like reliance on NGOs and limited government visits hinder service awareness. Overcoming these barriers, such as providing GMP cards and improving caregiver understanding, is crucial for enhancing program coverage (27, 32). Strengthening partnerships with NGOs and community organizations can promote caregivers' GMP service utilization (33, 34).

This study has three features that limit the conclusions that one can draw from the findings. Firstly, it was conducted in only a few subdistricts of rural Bangladesh, excluding urban areas, potentially compromising the representation of the country's overall GMP utilization scenario. Secondly, its cross-sectional design inhibits the establishment of causal relationships between caregivers' GMP utilization and associated factors. Thirdly, the absence of facility-based GMP utilization during the study period precludes the determination of predictors influencing caregivers' GMP service utilization.

Conclusion

This study contrasts home-based and facility-based GMP services in rural Bangladesh, favoring home-based options for caregivers with infants. The involvement of local NGOs underscores the importance of tailored, accessible approaches to meet caregivers' needs. Policymakers should prioritize integrating home-based GMP services within existing healthcare systems to optimize child health outcomes.

Acknowledgments

The study co-authors acknowledged the use of ChatGPT 3.5, developed by OpenAI, to edit the English language. The study team also acknowledges the role of two qualitative researchers, Ms. Ishrat Jahan and Dr. Ziaul Islam, for their contribution to qualitative data collection and analysis.

Funding statement: This work was supported by USAID under its Research for Decision Makers (RDM) program, award number: AID-388-A-17-00006. icddr,b is also grateful to the Governments of Bangladesh and Canada for providing core/unrestricted support.

Competing interests statement: None declared

Disclaimer: The funders had no role in the study design, data collection, analysis, or interpretation. The research was conducted independently of the funders, and the views expressed in this protocol are those of the authors.

Competing interests: None declared.

Reference

1. Ashworth A, Shrimpton R, Jamil K. Growth monitoring and promotion: review of evidence of impact. Matern Child Nutr. 2008;4 Suppl 1(Suppl 1):86-117.
2. Hossain M, Choudhury N, Adib Binte Abdullah K, Mondal P, Jackson AA, Walson J, et al. Evidence-based approaches to childhood stunting in low and middle-income countries: a systematic review. Arch Dis Child. 2017;102(10):903-9.
3. Singh A, Torres KA, Maharjan N, Shrestha J, Agbozo F, Abubakari A, et al. Learning from health system actor and caregiver experiences in Ghana and Nepal to strengthen growth monitoring and promotion. PLoS One. 2023;18(3):e0282807.
4. Bégin F, Elder L, Griffiths M, Holschneider S, Piwoz E, Ruel-Bergeron J, et al. Promoting Child Growth and Development in the Sustainable Development Goals Era: Is It Time for New Thinking? J Nutr. 2020;150(2):192-4.
5. Feleke FW, Adole AA, Bezabih AM. Utilization of growth monitoring and promotion services and associated factors among under two years of age children in Southern Ethiopia. PLoS One. 2017;12(5):e0177502.
6. Pollifrone MM, Cunningham K, Pandey Rana P, Philbin MM, Manandhar S, Lamsal KP, et al. Barriers and facilitators to growth monitoring and promotion in Nepal: Household, health worker and female community health volunteer perceptions. Matern Child Nutr. 2020;16(4):e12999.
7. Bilal SM, Moser A, Blanco R, Spigt M, Dinant GJ. Practices and challenges of growth monitoring and promotion in Ethiopia: a qualitative study. J Health Popul Nutr. 2014;32(3):441-51.

8. Charlton KE, Kawana BM, Hendricks MK. An assessment of the effectiveness of growth monitoring and promotion practices in the Lusaka district of Zambia. Nutrition. 2009;25(10):1035-46.
9. Hossain SMM, Duffield A, Taylor A. An evaluation of the impact of a US$60 million nutrition programme in Bangladesh. Health Policy and Planning. 2005;20(1):35-40.
10. Gwatkin DR, Bhuiya A, Victora CG. Making health systems more equitable. Lancet. 2004;364(9441):1273-80.
11. Fink G, Levenson R, Tembo S, Rockers PC. Home- and community-based growth monitoring to reduce early life growth faltering: an open-label, cluster-randomized controlled trial. Am J Clin Nutr. 2017;106(4):1070-7.
12. Riaz BK, Ali L, Ahmad SA, Islam MZ, Ahmed KR, Hossain S. Community clinics in Bangladesh: a unique example of public-private partnership. Heliyon. 2020;6(5).
13. Hossain M, Ferdous TE, Islam Z, Billah SM, Bulbul MMI, Rahman MM, et al. Protocol for a quasi-experimental study testing the effectiveness of strengthening growth monitoring and promotion in community clinics for improving the nutritional status of under-two children in rural Bangladesh. BMJ Open. 2019;9(10):e032458.
14. Panpanich R, Garner P. Growth monitoring in children. Cochrane Database Syst Rev. 2000(2):Cd001443.
15. Ministry of Health and Family Welfare DGoHS, Community Based Health Care programme, World Health Organization (WHO) Bangladesh. Independent evaluation of community-based health services in Bangladesh. Dhaka: Community Based Health Care (CBHC) Directorate General of Health Services (DGHS) Health Services Division, Ministry of Health and Family

Welfare Government of the People's Republic of Bangladesh and World Health Organization (WHO) Bangladesh; 2019.

16. Panpanich R, Garner P. Growth monitoring in children. Cochrane Database of Systematic Reviews. 1999(4).

17. National Institute of Population Research Training - NIPORT/Bangladesh, Mitra and Associates, ICF International. Bangladesh Demographic and Health Survey 2014. Dhaka, Bangladesh: NIPORT, Mitra and Associates, and ICF International; 2016.

18. de Onis M, Onyango A, Borghi E, Siyam A, Blössner M, Lutter C. Worldwide implementation of the WHO Child Growth Standards. Public Health Nutr. 2012;15(9):1603-10.

19. World Health Organization (WHO). WHO child growth standards: length/height-for-age, weight-for-age, weight-for-length, weight-for-height, and body mass index-for-age: methods and development. France: WHO; 2006.

20. Filmer D, Pritchett LH. Estimating wealth effects without expenditure data—or tears: an application to educational enrollments in states of India. Demography. 2001;38(1):115-32.

21. Ahmed SK. The pillars of trustworthiness in qualitative research. Journal of Medicine, Surgery, and Public Health. 2024;2:100051.

22. Newton-Lewis TA, Bahety G. Evaluating the effectiveness of Community Health Worker home visits on infant health: A quasi-experimental evaluation of Home Based Newborn Care Plus in India. J Glob Health. 2021;11:04060.

23. Sudfeld CR, Bliznashka L, Ashery G, Yousafzai AK, Masanja H. Effect of a home-based health, nutrition and responsive stimulation intervention and conditional cash transfers on

child development and growth: a cluster-randomised controlled trial in Tanzania. BMJ Glob Health. 2021;6(4).

24. Teklemuz N, Sisay M, Baffa LD, Mengistu B, Atenafu A. Utilization of growth monitoring and promotion services among children younger than 2 years in West Armachiho district, Northwest Ethiopia. Frontiers in Public Health. 2023;11.

25. Agbozo F, Colecraft E, Jahn A, Guetterman T. Understanding why child welfare clinic attendance and growth of children in the nutrition surveillance programme is below target: lessons learnt from a mixed methods study in Ghana. BMC Nurs. 2018;17:25.

26. Tekle M, Tariku B, Alagaw A, Zerihun E, Bekele HW. Exploring Reasons for Low Attendance of Mothers to Growth Monitoring and Promotion Program at Loka Abaya District, Southern Ethiopia: Exploratory Qualitative Study. J Nutr Metab. 2019;2019:3510649.

27. Nguyen PH, Khương LQ, Pramanik P, Billah SM, Menon P, Piwoz E, et al. Effective coverage of nutrition interventions across the continuum of care in Bangladesh: insights from nationwide cross-sectional household and health facility surveys. BMJ Open. 2021;11(1):e040109.

28. Shonchoy AS, Akram AA, Khan M, Khalid H, Mazhar S, Khan A, et al. A Community Health Worker–Based Intervention on Anthropometric Outcomes of Children Aged 3 to 21 Months in Urban Pakistan, 2019–2021. American Journal of Public Health. 2023;113(1):105-14.

29. Nisbett N, Davis P, Yosef S, Akhtar N. Bangladesh’s story of change in nutrition: Strong improvements in basic and underlying determinants with an unfinished agenda for direct community level support. Global Food Security. 2017;13:21-9.

30. Roberfroid D, Pelto GH, Kolsteren P. Plot and see! Maternal comprehension of growth charts worldwide. Tropical Medicine & International Health. 2007;12(9):1074-86.
31. Tesfa M, Gonete KA, Chane Y, Yohannes S. Growth Monitoring Practice and Associated Factors Among Health Professionals at Public Health Facilities of Bahir Dar Health Centers, Northwest Ethiopia, 2021. Pediatric Health Med Ther. 2022;13:195-215.
32. Sulley I, Abizari AR, Ali Z, Peprah W, Yakubu HG, Forfoe WW, et al. Growth monitoring and promotion practices among health workers may be suboptimal despite high knowledge scores. BMC Health Serv Res. 2019;19(1):267.
33. El Arifeen S, Christou A, Reichenbach L, Osman FA, Azad K, Islam KS, et al. Community-based approaches and partnerships: innovations in health-service delivery in Bangladesh. The Lancet. 2013;382(9909):2012-26.
34. Marek T, Diallo I, Ndiaye B, Rakotosalama J. Successful contracting of prevention services: fighting malnutrition in Senegal and Madagascar. Health Policy Plan. 1999;14(4):382-9.

 Click here to access/download;Figure;Figure_1.tif

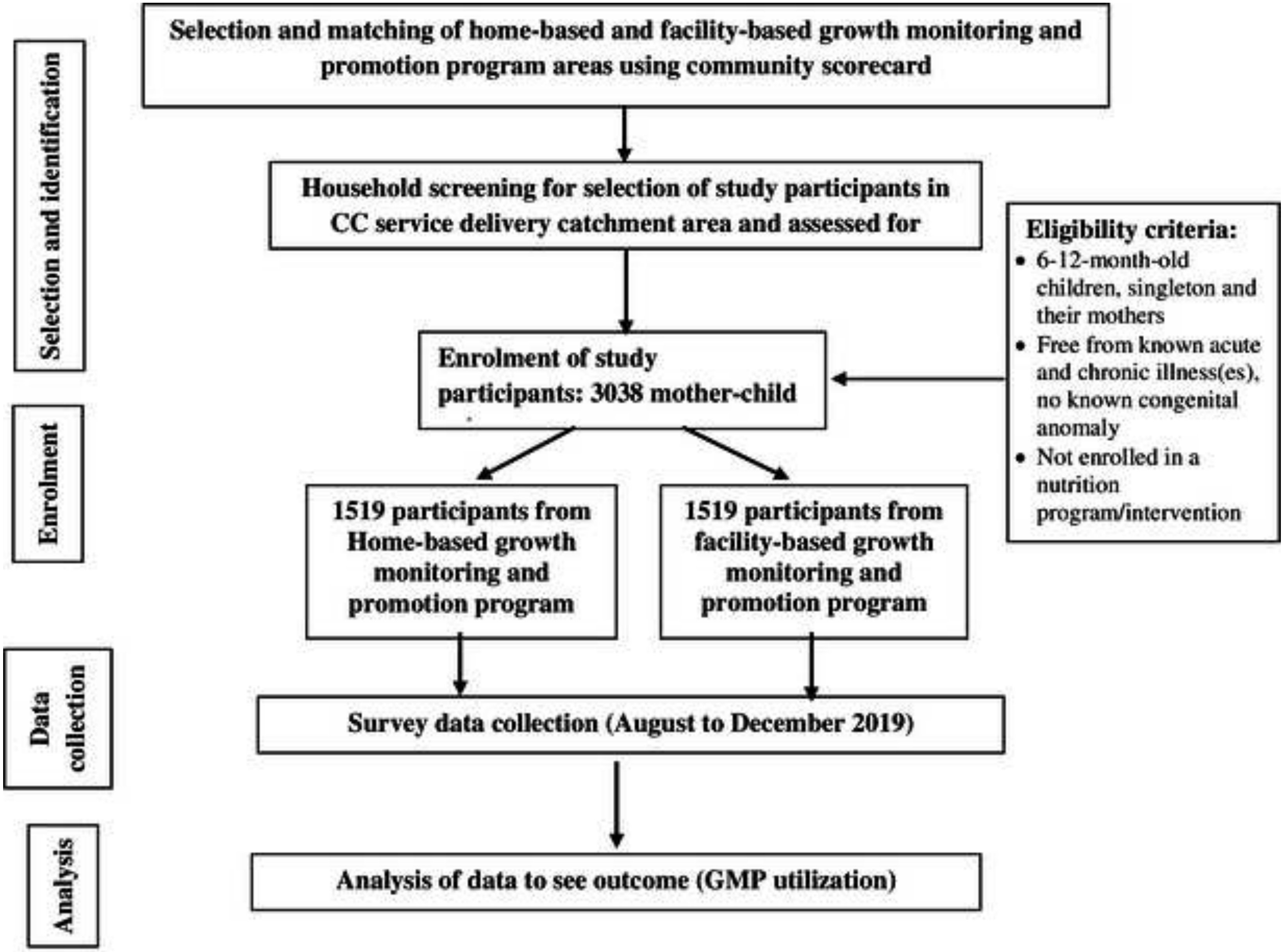

Figure 1: Participants flow chart
