# Supplementary material for: Growth monitoring and promotion program services utilization patterns between home-based and facility-based delivery methods: A comparative analysis
Source: PLoS One. 2025 Jun 5;20(6):e0324918. doi: 10.1371/journal.pone.0324918 (PMC12140421; doi:10.1371/journal.pone.0324918)
Supplement: Attachment — Submitted filename: Response to Reviewers.docx [file pone.0324918.s010.docx]

6 February 2025

Zeheng Wang
Academic Editor
PLOS ONE

California, USA

RE: PONE-D-24-20319: Growth monitoring and promotion program services utilization patterns between home-based and facility-based delivery methods: a comparative analysis

Dear Academic Editor,

We thank you for your response to our manuscript. Please find attached a revised version, in response to your invitation to resubmit the paper to PLOS ONE if we felt that we could satisfactorily address the concerns of the reviewers. We have substantially modified the manuscript, taking into account the comments from the editorial office and the four reviewers.

Our response to the comments is presented using the following structure: The original editorial / referee comment is copied into this letter in italics. Our response to the comment is written below in normal text format. In case a revision to the manuscript text has been made, we give the appropriate page number(s). Changes in the text have been indicated using yellow text highlighting in the manuscript and copied into this letter in the same format. The highlighted text file has been uploaded into your electronic system as a “Revised Manuscript with Track Changes”.

**Responses to the comments made by the academic editor:**

*1.Please ensure that your manuscript meets PLOS ONE's style requirements, including those for file naming. The PLOS ONE style templates can be found at*

[*https://journals.plos.org/plosone/s/file?id=wjVg/PLOSOne_formatting_sample_main_body.pdf*](https://journals.plos.org/plosone/s/file?id=wjVg/PLOSOne_formatting_sample_main_body.pdf) *and*

[*https://journals.plos.org/plosone/s/file?id=ba62/PLOSOne_formatting_sample_title_authors_affiliations.pdf*](https://track.editorialmanager.com/CL0/https:%2F%2Fjournals.plos.org%2Fplosone%2Fs%2Ffile%3Fid=ba62%2FPLOSOne_formatting_sample_title_authors_affiliations.pdf/1/010f01942c9445eb-8d5fc183-f7c2-4b3d-b852-b898a6a1b8dd-000000/vaM6EGOsCJhWDadMs_lucRlv0A8R8wQXt-YNbKOd_vU=191)

Our response: Thank you for your suggestion. We reformatted the manuscript by following the PLOS ONE’s style requirements including those for file naming.

*2. Please note that funding information should not appear in any section or other areas of your manuscript. We will only publish funding information present in the Funding Statement section of the online submission form. Please remove any funding-related text from the manuscript.*

Our response: We removed funding information from the manuscript as suggested.

*3. We note that you have indicated that there are restrictions to data sharing for this study. PLOS only allows data to be available upon request if there are legal or ethical restrictions on sharing data publicly. For more information on unacceptable data access restrictions, please see* [*http://journals.plos.org/plosone/s/data-availability#loc-unacceptable-data-access-restrictions*](http://journals.plos.org/plosone/s/data-availability#loc-unacceptable-data-access-restrictions)*.*

*Before we proceed with your manuscript, please address the following prompts:*

*a) If there are ethical or legal restrictions on sharing a de-identified data set, please explain them in detail (e.g., data contain potentially identifying or sensitive patient information, data are owned by a third-party organization, etc.) and who has imposed them (e.g., a Research Ethics Committee or Institutional Review Board, etc.). Please also provide contact information for a data access committee, ethics committee, or other institutional body to which data requests may be sent.*

*b) If there are no restrictions, please upload the minimal anonymized data set necessary to replicate your study findings to a stable, public repository and provide us with the relevant URLs, DOIs, or accession numbers. For a list of recommended repositories, please see*

[*https://journals.plos.org/plosone/s/recommended-repositories*](https://journals.plos.org/plosone/s/recommended-repositories)*. You also have the option of uploading the data as Supporting Information files, but we would recommend depositing data directly to a data repository if possible.*

*We will update your Data Availability statement on your behalf to reflect the information you provide.*

Our response: We would like to share the data as Supporting information file reads as (line 596):

S3 File. Study data set

*4. We note you have included a table to which you do not refer in the text of your manuscript. Please ensure that you refer to Table 4 in your text; if accepted, production will need this reference to link the reader to the Table.*

Our response: We now include Table 4 inside the text (lines 306, 314).

“Four major barriers were identified as reasons for caregivers' underutilization of GMP services. Among these, caregivers' lack of knowledge about the availability of GMP services at the nearest health facility was a significant barrier in both areas (Table 4).”

More caregivers from home-based GMP service areas reported closure of health facilities/absence of service providers (1%) and being preoccupied with household work (4%) as significant barriers to accessing and utilizing GMP services, compared to facility-based GMP service areas (0.2% and 0.3%, respectively) (Table 4).”

**Responses to the comments made by reviewer # 1:**

*Reviewer #1: The study investigated the use of growth monitoring and promotion services in rural Bangladesh, comparing home-based and facility-based programs. The primary goal was to understand the factors influencing caregiver engagement with GMP services. The article's manuscript and statistical presentation of some of the major data appear to be relevant to a community-based study, particularly when considering the use of growth monitoring and promotion services. Its presentation and findings are satisfactory enough for publication. Therefore, I recommend acceptance of this manuscript, but with minor corrections.*

1. *The manuscript has used abbreviations inconsistently.
   Line No. 14: Replace the GMP abbreviation with Growth Monitoring and Promotion (GMP).*

Our response: Thank you for pointing this out. We provided the full form of GMP (lines 14-15).

“In Bangladesh, utilization of government health facility-based growth monitoring and promotion (GMP) services is notably low, prompting non-governmental organizations (NGOs) to collaborate with the government to enhance GMP service utilization through home-based delivery.”

*2. Line No. 303: Growth Monitoring and Promotion Should be replaced with the GMP abbreviation.*

Our response: We replaced the full form with GMP abbreviation, as suggested (line 349).

“The study aimed to assess caregivers' knowledge, perception, utilization, and barriers to accessing GMP services in rural Bangladesh, comparing home-based and facility-based settings.”

*3. Line Nos. 114, 259, and 309: the abbreviations GM for Growth Monitoring may not be required.*

Our response: We kept the full form “growth monitoring” and removed GM abbreviation, as suggested (lines 137, 301, 302).

**Responses to the comments made by reviewer # 2**

*Reviewer #2: In addition to comments and feedbacks provided in reviewed manuscript attached, I want to mention that operational definitions viz wealth quintile, service utilization index etc should be added in method section. Some discussion is required like why facility based GMP utilization is almost zero, why home based service utilization is also lower than expectation ( around 40% only) etc*

Our response: Thank you so much for your valuable comments and guidance. We addressed all your comments inside the manuscript, comment boxes as suggested and highlighted them.

- The definition of wealth index revised text in the methods section reads as follows (lines 188-190):

“A wealth index was constructed from household assets using a composite score to classify socioeconomic status (19). Households were grouped into three categories: rich, middle, and poor, reflecting relative differences in asset ownership.”

- We added the term “operational definition” for service utilization index in methods section reads as follows (line 157):

“Operational definition: Caregivers' GMP service utilization was defined as whether they received GMP services at home or went to the facility for the service even once during the child’s lifetime during the survey”

- We provided the reason in discussion section on “*why facility based GMP utilization is almost zero, why home based service utilization is also lower than expectation*” reads as follows (lines 359-368):

“The stark contrast in GMP service utilization, with home-based services at 40% and facility-based services at 0%, underscores critical barriers in accessing and delivering care. While home-based services perform better, their utilization remains lower than expected due to irregular or insufficient visits by health workers, limited caregiver awareness of GMP’s importance, and competing priorities or time constraints (21). In contrast, the zero utilization of facility-based GMP highlights additional challenges such as limited accessibility to health facilities, lack of awareness or trust in facility-based services, and caregivers’ preference for more convenient or culturally appropriate home-based interventions (22). Moreover, the absence of facility-based utilization may reflect systemic gaps in infrastructure, staffing, and health system outreach efforts (6).

**Responses to the comments made by reviewer # 3:**

*Reviewer #3: This is a very important and little studied topic.

Some points that I believe would be worth discussing further:

1. The study is limited to rural sub-districts, not covering urban scenarios that may present different barriers and contexts.*

Our response: We agree with your concern. We already mentioned this as limitation in the discussion section (lines 441-443):

“Firstly, it was conducted in only a few subdistricts of rural Bangladesh, excluding urban areas, potentially compromising the representation of the country's overall GMP utilization scenario.”

2. *The absence of longitudinal data prevents the assessment of long-term impact.*

Our response: Yes, we agree with your comment and added the following sentence in discussion section, read as follows (lines 444-445):

“Thirdly, the absence of longitudinal data prevents the assessment of long-term impact.”

*3. The gap in the provision of GMP cards in government facilities highlights a structural problem that could be discussed more strongly.*

Our response: We edited as suggested in the discussion section, read as follows (lines 407-414):

“The insufficient supply of GMP cards in government facilities significantly contributes to their non-utilization and highlights a critical structural issue within the health system [26, 31, 32]. This shortfall stems from disrupted supply chains, limited funding, or inadequate prioritization of GMP services, ultimately hindering caregivers’ ability to track and engage with child growth monitoring [32]. Barriers like health facility closures and household responsibilities also explain low utilization rates [6]. To address this, systemic reforms are needed to ensure a reliable supply of GMP cards, as they are essential tools for promoting effective service delivery and improving child health outcomes.”

4. *Specific strategies to better integrate NGO initiatives into the public health system could be better described.*

Our response: We now added strategies that could help to integrate NGO initiatives into the public health system in discussion section, read as follows (lines 422-427):

“To improve GMP utilization, specific strategies for integrating NGO initiatives into the public health system should focus on strengthening coordination and resource sharing. This includes joint training of staff, aligning service delivery goals, and establishing referral and follow-up mechanisms between home-based and facility-based services [33, 34]. Such integration can bridge service gaps, enhance caregiver awareness, and improve access to GMP services.”

5. Qualitative analysis presents data that could be further explored.

Response: Our response: We have added information from qualitative analysis in the results section (lines 320-324):

“Caregivers from facility-based GMP areas never received GMP cards because of lack of supply of GMP cards in CCs from the government. Interviews with government stakeholders revealed the lack of allocation of government funds to print and supply GMP cards to the CCs during the study period. The NGO area caregivers got GMP cards as the NGO printed out and distributed government approved GMP cards using their own funds.”

**Responses to the comments made by reviewer # 4:**

*Reviewer #4: This study evaluates and compares utilization patterns and barriers to caregiver engagement in Growth Monitoring and Promotion (GMP) services delivered via home-based (3 sub-districts) and facility-based (3 sub-districts) mechanisms in Mymensingh district, Bangladesh. The research aims to identify efficient and effective public health delivery models in a resource-limited setting of a low- and middle-income country. While well-written, the proposal could benefit from addressing the following comments:

• Title: The primary focus of the study is to assess and compare GMP service utilization and identify the barriers; however, the title focuses on the caregiver utilization. Consider the following alternative titles:
o "Disparities in Utilization and Barriers to Caregiver Engagement in Growth Monitoring and Promotion Services: A Comparative Study of Home-Based and Facility-Based Programs."
o "Disparities and Barriers in Growth Monitoring and Promotion Services: Home-Based vs. Facility-Based Programs."
o "Growth Monitoring and Promotion Program Services Utilization Patterns Between Home-Based and Facility-Based Delivery Methods: A Comparative Analysis."
o "Comparative Utilization Patterns of Growth Monitoring and Promotion Services: Home-Based Versus Facility-Based Delivery Methods."*

Our response: Thank you for suggesting 4 options for a title. We picked the 3rd one and revised our title as “Growth Monitoring and Promotion Program Services Utilization Patterns Between Home-Based and Facility-Based Delivery Methods: A Comparative Analysis."

*• Page 2 Line 14: Expand the abbreviation GMP at its first use in the abstract.*

Our response: We edited as suggested, read as follows (line 14-15):

“Background: In Bangladesh, utilization of government health facility-based growth monitoring and promotion (GMP) services is notably low, prompting non-governmental organizations (NGOs) to collaborate with the government to enhance GMP service utilization through home-based delivery.”

*• Page 3 Line 38: “Conclusion: GMP service utilization remains low in some parts of rural Bangladesh”.*

Our response: We revised the sentence and now read as following (line 38):

“Conclusion: GMP service utilization remains low in rural Mymensingh district of Bangladesh.”

*o Compare and contrast the sociodemographic characteristics of the Mymensingh district with other similar districts and national metrics in Bangladesh.*

Our response: We added the information, read as following (lines 94-95):

“Mymensingh is similar to other Bangladeshi districts with its predominantly rural, agrarian population, along with comparable literacy rates and demographic patterns.”

*o Provide a clear rationale for selecting Mymensingh for this comparative study, apart from the presence of NGO services.*

Our response: This following information has been added in the methods section (lines 95-98):

“We selected the Mymensingh district based on the following criteria: (A) time of implementation of the programme matching with the study timeline; (B) staff and logistics in CCs supported by NGO; and (C) an adequate number of CCs in subdistricts to satisfy the sample size [13].”

*o Analyze and compare the characteristics of sub-districts offering home-based versus facility-based GMP services.*

Our response: We added the information in the results section reads as follows (lines 268-269) and uploaded the table as supplementary file:

“The socio-demographic characteristics of the six sub-districts in 2 GMP areas were mostly comparable (S1 file).”

*• Page 6 Line 104-108:
o Justify the exclusion of children with acute and chronic illnesses, stunting, wasting, underweight, or those not enrolled in similar programs, as these groups might lack access to or awareness of GMP services.*

Our response: We revised as suggested, read as follows (lines 125-128):

“Eligibility criteria encompassed children free from known acute/chronic illnesses, severe stunting, wasting, or underweight due to ethical obligation as the study would not provide treatment or management, not enrolled in similar programs, and whose caregivers provided consent.”

*o Indicate the proportion of children excluded and assess the impact on study findings and GMP service utilization metrics.*

Our response: Edited as suggested in the results section (lines 251-253):

“We excluded children during household screening which is about 16% (n=291) in intervention area and 7% (n=122) in control area.”

In discussion section (lines 379-381):

“We did not collect information from excluded children and thus could not assess the impact on study findings and GMP service utilization matrix”

*o Discuss potential selection biases introduced by these exclusions, the rate of GMP service utilization among excluded children.*

Our response: Edited as suggested in discussion section (lines 372-373, 377-382):

“………exclusion of children with known chronic illness, severely malnourished children and twins, and the relatively brief duration of the study.” …… Our study included only apparently healthy, disease-free children, which might introduce a selection bias and limit the generalizability of the findings to children with underlying health conditions. We did not collect information from excluded children and thus could not assess the impact on study findings and GMP service utilization matrix. We excluded twins to reduce potential information bias.”

*o Explain the bias introduced by including twins in the study and its implications for the results.*

Our response: Our sincere apologies for the confusion. The twins were already excluded during screening, and we now have clarified this in the methods and in the discussion section, reads as follows (lines 132, 395-396):

“We excluded twins during screening to reduce potential information bias.”

*• Page 7 Line 111-112: “The components of GMP are intended, not documented, actions.” Rewrite the sentence with clarity.*

Our response: : Edited as suggested and read as follows (lines 136-138):

“The components of GMP outlined in this section represent the ideal elements of a GMP program as it should be, rather than reflecting the practical realities of implementation. ”
*• Page 7 Line 115: Could the poor health outcomes of excluded children be attributed to a lack of access to GMP services?*

Our response: Edited as follows in methods section (lines 138-139):

“If health workers identified a child was acutely ill or not growing well during GMP he/she should refer the child to nearest medical/treatment center (14).”

• *Page 9 Line 163: What proportion of responses were identified as incorrect?*

Our response: Added as suggested in results section and reads as follows (lines 279-281):

“A total of 2,791 (91.8%) out of 3,038 responses from mothers were incorrect; Among them intervention, n=1,274 (83.8%) and control, n=1,517 (99.8%) responses were incorrect.”

*• Page 10 Line 190-192: Were questionaries in Bangla validated? If yes, please state it in the paper.*

Our response: We provided the information inside the methods section (lines 216-217):

“The Bangla version of the questionnaire was validated before the interview through translation and back-translation (13).”

*• Page 10 Line 193: The abbreviation CC (Community Clinic) was already introduced earlier in line 119.*

Our response: We edited as suggested (line 218).

*• Page 11 Line 207-208: “Only some relevant participants were interviewed for the qualitative part.” Define "relevant participants" in the qualitative analysis and explain how they differ from those in the quantitative survey. Show sociodemographic characteristics of both groups.*

Our response: We edited as suggested and added the information in methods section (lines 234-236):

“The qualitative participants were selected in a purposive manner from the program areas to supplement the quantitative findings. However, we did not collect their detailed socio-demographic characteristics to make a comparison.”

*• Page 11 Line 208-209: “The number of qualitative interviews was decided following an iterative process of achieving information saturation.” Explain the ‘iterative process’ and ‘information saturation’ for non-technical readers.*

Our response: We provided this information inside the manuscript which reads as follows (lines 236-240):

“The number of qualitative interviews was determined through an iterative process, where data from each round of interviews were analyzed to identify emerging themes. Interviews continued until information saturation was reached—that is, when no new insights were gained, indicating that key themes had been thoroughly explored.”

*• Page 12 Line 224-225: Expand the abbreviation LAZ, WAZ, and WHZ at their first use.*

Our response: We added the information as suggested, which reads as follows (lines 258-261):

“ Children's length-for-age Z-score (LAZ) and weight-for-age Z score (WAZ) was comparable among the groups. Children from home-based GMP area had significantly lower weight-for-length/height Z-score (WHZ) compared to facility-based area children”

*• Page 14 Line 235: It appears unrealistic that* *only 0.4% caregiver would have heard of GMP cards. Please provide government data and other research that have reported GMP card awareness in the facility-based GMP services in Bangladesh.*

Our response: Edited as suggested in the discussion section (lines 353-356):

“This explains why only 0.4% of caregivers would have heard of GMP cards in facility based GMP areas. However, we could not compare these results with government or other research data, since our study is the first to report this and there is no such information available.”

*• Page 14 Line 240-241:
o The reported awareness (0.4%) of GMP cards seems unrealistically low. Provide government and other research data for comparison, particularly for facility-based GMP services in Bangladesh.*

Our response: We have already provided the response to this comment.

*o Additionally, clarify the definition of “beneficial” and address the absence of perceived benefit in facility-based sub-districts using government and other research data.*

Our response: Edited as suggested in results section (lines 283-284):

“The meaning of the word “beneficial” (“*valo/upokari”* in Bangla) was not explained to the respondent, but it was left for them to judge”

In discussion section, we edited as follows (lines 391-393):

“This study is first to report on the absence of perceived benefit in facility-based sub-districts, which is unique as there is no government or research data available.”

• Table 1: Were sociodemographic characteristics statistically different? Include P-value.

Our response: We added p-values in table 1 as suggested, read as follows (line 270):

**Table 1.** **Caregiver's sociodemographic characteristics**

| **Characteristic** | | **Home-based GMP**  N = 1,519 | **Facility-based GMP**  N = 1,519 | **p-value** |
| --- | --- | --- | --- | --- |
| Child age in months, mean (SD) | | 9.9 (1.7) | 9.7 (1.8) | 0.065 |
| Child's weight-for-age Z-score (WAZ), mean (SD) | | -1.1 (1.1) | -1.1 (1.1) | 0.555 |
| Child's length-for-age Z-score (LAZ), mean (SD) | | -1.3 (1.1) | -1.4 (1.1) | 0.190 |
| Child's weight-for-length Z-score (WHZ), mean (SD) | | -0.48 (0.03) | -0.40 (0.03) | 0.043 |
| Proportion of boys, n (%) | | 760 (50.0) | 789 (51.9) | 0.293 |
| Mother's age in years, mean (SD) | | 26.1 (5.8) | 25.6 (5.9) | 0.011 |
| Mother's education in years, mean (SD) | | 6.1 (3.6) | 6.1 (3.6) | 0.717 |
| Mother's occupation, n (%) | |  |  |  |
| Housewife/homemakers | | 1,474 (97.0) | 1,487 (97.9) | 0.133 |
| Fathers' education in years, mean (SD) | | 7.4 (4.5) | 6.9 (3.4) | 0.002 |
| Religion, n (%) | |  |  |  |
|  | Islam | 1,494 (98.4) | 1,478 (97.3) | 0.046 |
|  | Hinduism/Buddhism/Christian | 25 (1.6) | 41 (2.7) |  |
| Improved toilet facility, n (%) | | 1,402 (92.3) | 1,435 (94.5) | 0.016 |
| Improved source of drinking water, n (%) | | 1,514 (99.7) | 1,515 (99.7) | 0.739 |
| Wealth index, n (%) | |  |  |  |
|  | Poor | 667 (44.0) | 577 (38.0) | 0.002 |
|  | Middle | 430 (28.3) | 461 (30.3) |  |
|  | Rich | 420 (27.7) | 481 (31.7) |  |

We edited the findings in the results section, read as follows (lines 258-268):

“Children's length-for-age Z-score (LAZ) and weight-for-age Z score (WAZ) was comparable among the groups. Children from home-based GMP area had significantly lower weight-for-length/height Z-score (WHZ) compared to facility-based area children (mean WHZ: -0.48 versus -0.4, p=0.04). Mother’s age (home-based vs. facility-based: 26.1 vs 25 years) and father’s years of education (home-based vs. facility-based: 7.4 vs 6.9 years) were significantly higher in home-based GMP area compared to facility-based GMP area (p=0.01 and 0.002 respectively) (Table 1). Approximately 98% of household members in both areas identified as Muslim, with 94% and 92% having improved toilets in home-based and facility-based GMP service areas, respectively (p=0.016) (Table 1). More (44%) caregivers in home-based GMP areas were from poor families compared to facility-based GMP areas (38%) (p=0.002) (Table 1).”

In the discussion section, we added a paragraph to address the issue, read as follows (lines 428-439):

“The study highlights the critical role of accessibility and convenience in influencing GMP utilization patterns. Home-based GMP services, despite their availability, exhibited low utilization rates, suggesting that accessibility alone may not drive engagement. Factors such as awareness, perceived benefits, and trust in service providers likely played a role in shaping caregivers' decisions. Additionally, the study acknowledges that the two populations (FGMP and HGMP) had some baseline differences beyond just the type of GMP received. These initial differences may have influenced the outcomes observed, potentially confounding the effects attributed solely to GMP type. While our analysis aimed to account for these variations, the findings should be interpreted with caution, considering that factors could have contributed to the observed differences between groups. Future research should explore these factors further to provide a more comprehensive understanding of GMP utilization dynamics.”

*• Figure 1: “Selection and matching…”. Was matching performed? If matching was performed between home-based and facility-based cohorts, provide methodological details.*

Our response: We matched the areas based on some socio-demographic characteristics. We now provided the details in the methods section with reference (lines 104-119):

“The home and facility-based areas are matched based on some socio-demographic characteristics to avoid selection bias (13). Initially, study-specific general information was collected on each CC and its catchment area through discussions with three groups of residents: (1) Community health care providers (CHCPs), (2) mothers/caregivers of under-two children, and (3) members of CC-associated community group (CG). Discussions focused on: (1) GMP service availability, (2) availability of other nutrition services at the CCs for women and children, (3) components of GMP provided by CHCP, and (4) sociodemographic status of people living in the CC catchment area. One point was given to a response if it contains more than or equal to 90% response rate (positive or negative), and less than 90% response got zero, totaling to a maximum of 5 points for a given CC. All the points were then summed to generate a combined score for each CC. Majority of the intervention area CCs (more than 90%) scored 3 out of 5. In the next stage, due to the similarities of scores, 30 intervention CCs were selected by computer-generated simple random sampling from the total 72 CCs in the intervention subdistricts. Similar scores were generated for each control CC. The 30 control CCs were then individually matched with the 30 intervention CCs based on the closest scores. Thus, the intervention-to-control CC ratio was 1:1.”

*• Add the following supplementary materials:
o Include details of statistical analyses, such as normality tests and full models with covariates.
o Add the questionnaire (with English translation) to aid reader comprehension of questions and outcome metrics.*

Our Response: We added all suggested materials as “Supporting file” (lines 594-596):

S1 File. Details on statistical analysis

S2 File. Study questionnaire in English

S3 File. Study data set

We hope that in its present form the manuscript is acceptable for publication in PLOS ONE. The material has not and will not be offered elsewhere for possible publication, as long as it is under PLOS ONE journal consideration.

Sincerely,

Dr. Muttaquina Hossain

Associate Scientist

Nutrition Research Division, icddr,b

Email: [muttaquina@icddrb.org](mailto:muttaquina@icddrb.org); [muttaquina@gmail.com](mailto:muttaquina@gmail.com)

Mobile: +88-01923758956
