# Supplementary material for: Growth monitoring and promotion program services utilization patterns between home-based and facility-based delivery methods: A comparative analysis
Source: PLoS One. 2025 Jun 5;20(6):e0324918. doi: 10.1371/journal.pone.0324918 (PMC12140421; doi:10.1371/journal.pone.0324918)
Supplement: Attachment — Submitted filename: Peer review PLOS ONE.docx [file pone.0324918.s008.docx]

February 24th, 2025

Dear Academic Editor,

This is an important field of study, and the article merits publication. The authors appear to have comprehensively responded to the feedback from all four reviewers and have made the required revisions. Here are my comments:

1. I suggest including the word "Barriers" among the keywords in the abstract.

2. The number of sources cited throughout the article is insufficient. I recommend adding more current sources throughout the various sections of the article.

3. There are instances where the same sources are repeated within the same paragraph and occasionally in other parts of the article. I suggest significantly diversifying the sources used.

4. It's crucial to include up-to-date references from studies published within the last years.

5. Has the semi-structured questionnaire, which was pretested, been validated by content experts? Was the measurement instrument developed based on scientific literature? Has it undergone translation from Bangladeshi to English and then back to Bangladeshi?

6. Are researchers completely confident that no other study has ever reported a lack of GMP use in facilities—a situation that is unique to this setting and contrasts with findings from existing LMIC GMP programs? I recommend rephrasing this sentence in a more modest and careful manner.

7. The conclusions section is limited to just one paragraph, making it excessively concise and in need of substantial expansion. Furthermore, it offers only one sentence of recommendations for policymakers focused on shaping public health strategies. It points out that simply prioritizing among the nation's decision-makers for the integration of home-based GMP services is insufficient.

Best regards,

Tamir Velner
