# Supplementary material for: Growth monitoring and promotion program services utilization patterns between home-based and facility-based delivery methods: A comparative analysis
Source: PLoS One. 2025 Jun 5;20(6):e0324918. doi: 10.1371/journal.pone.0324918 (PMC12140421; doi:10.1371/journal.pone.0324918)
Supplement: Attachment — Submitted filename: Manuscript-reviewed by SV.docx [file pone.0324918.s009.docx]

**Title:** Growth monitoring and promotion program services utilization patterns between home-based and facility-based delivery methods: a comparative analysis

**Author:** Muttaquina Hossain^1*^, Md. Ahshanul Haque^1^, A.S.G. Faruque^1^, Tahmeed Ahmed^1, 2^, Ulla Ashorn^3^, Per Ashorn^3,4^

**Affiliations:**

^1^ Nutrition Research Division, icddr,b, Mohakhali, Dhaka, Bangladesh

^2^ James P Grant School of Public Health, BRAC University, Mohakhali, Dhaka, Bangladesh

^3^ Center for Child, Adolescent and Maternal Health Research, Faculty of Medicine and

Health Technology, Tampere University, Tampere, Finland

^4^ Department of Paediatrics, Tampere University Hospital, Tampere, Finland

*Corresponding author: E-mail: [muttaquina@icddrb.org](mailto:muttaquina@icddrb.org); [muttaquina@gmail.com](mailto:muttaquina@gmail.com) (MH)

# **Abstract**

Background: In Bangladesh, utilization of government health facility-based growth monitoring and promotion (GMP) services is notably low, prompting non-governmental organizations (NGOs) to collaborate with the government to enhance GMP service utilization through home-based delivery. Despite this, there is limited information available on home-based GMP service utilization. This study takes a unique approach by investigating the utilization of GMP services between home-based and facility-based programs and identifying key factors and barriers to caregiver engagement with GMP services in rural Bangladesh.

Methods: A comprehensive descriptive mixed-method study was conducted across six sub-districts over five months. Three sub-districts with home-based GMP services provided by NGOs were compared with three neighboring sub-districts offering government facility-based GMP services. A total of 3038 mothers and children under one year old were included. GMP utilization was defined as caregivers receiving GMP services at least once in the child’s lifetime. Quantitative surveys and qualitative approaches were used for data collection. Multiple logistic regression was employed to determine statistically significant associations between GMP utilization and independent variables at p-value <0.05 and adjusted risk ratio (ARR) values with a 95% confidence interval (CI). Thematic analysis of qualitative data was performed. Results were triangulated across sources.

Results: Home-based GMP services exhibited higher utilization rates, with more children receiving weight and length measurements and caregivers receiving counseling than facility-based services (40% vs. 0% utilization, respectively). Caregivers' utilization of GMP services in home-based areas was positively influenced by their knowledge of GMP or GMP cards (Adjusted risk ratio, ARR: 37.4, 95% confidence interval, 95% CI: 17.8,78.5) and their involvement with an NGO, association, or health program (ARR: 1.3, 95% CI: 1.1, 1.5). Lack of caregiver interest and awareness of GMP services emerged as significant barriers.

Conclusion: GMP service utilization remains low in rural Mymensingh district of Bangladesh. Tailored healthcare solutions addressing caregivers' needs are crucial to improving GMP program coverage and utilization rates.

Key Words: Growth monitoring and promotion, Children, Service Utilization, Program, Bangladesh

# **Introduction**

*The GMP program is designed to include regular check-ups for child growth and happens every three months at health facilities. In an ideal GMP program Health workers are advised to counsel mothers and caregivers on their children's nutrition, measure the weight, length, or mid-upper arm circumference (MUAC) of all under-2-year-old children, and plot the results on a growth monitoring chart in the GMP card. If a health worker identifies a child as acutely ill or not growing well during GMP, he/she should refer the child to the nearest medical/treatment center [14].”* Growth Monitoring and Promotion (GMP) represent integral components of preventive and promotional endeavors, employing recurrent measurements and analyses of child growth to discern growth faltering. This approach aims to foster communication and interaction with caregivers, promoting optimal child development [1]. The introduction of the promotion component by UNICEF in the mid-1980s augmented the concept, rendering GMP a vital facet of nutrition programs beneficial to effective malnutrition management in children within low- and middle-income country (LMIC) settings [2].

The effectiveness of GMP programs depends on factors like program coverage, caregiver interactions with healthcare providers, health worker performance, facility readiness, community engagement, appropriateness of delivery platform, and sociocultural contexts, stressing the need for optimal resource mobilization [1, 3]. Primarily implemented through health facilities in LMICs, research on GMP programs has predominantly focused on identifying deficiencies in facility-level readiness and supply-side inadequacies [1, 4-6]. These investigations often neglect the crucial assessment of acceptability and feasibility from the perspectives of caregivers and communities, along with their sociocultural contexts. The effectiveness of facility-based GMP programs in LMICs in improving caregiver utilization and reducing child undernutrition remains uncertain [5, 7-9].

To bridge this gap in facility-based GMP service delivery, certain LMICs in Africa and Asia have adopted community and home-based platforms for GMP service delivery [1]. The effectiveness of these decentralized approaches hinges upon factors such as the communication and counseling skills of health workers, the motivation and willingness of caregivers to engage with the service, and the accessibility of appropriate referrals and interventions [10]. However, despite the promise of these alternatives, most home/community-based GMP services remain isolated and lack linkage with the broader health system, posing challenges to sustainability and scalability [1]. Consequently, the outcomes of home-based GMP services exhibit variability across diverse contexts and regions [11].

In the ongoing debate surrounding the most effective delivery platform for GMP services in Low- and Middle-Income Countries (LMICs) with limited resources, This study aims to delve into caregiver GMP service utilization across both home-based and facility-based settings. The primary objectives are twofold: firstly, to ascertain whether disparities exist in GMP service utilization between home-based and facility-based programs, and secondly, to pinpoint and analyze the principal barriers hindering caregiver engagement with GMP services in these respective contexts. Qualitative approaches are applied to enhance the quantitative survey findings. By comprehensively examining the dynamics of service utilization and the multifaceted factors influencing caregiver participation, valuable information will be provided to researchers, program developers, public health practitioners, and policymakers. Through a rigorous analysis of these anticipated outcomes, evidence-based decision-making processes will be fostered, ultimately enhancing the utilization of GMP services for caregivers and improving healthcare outcomes in LMICs.

# **Materials and methods**

## **Study design, period, and site**

A community-based cross-sectional study employing a mixed-method approach was conducted in six rural sub-districts of Bangladesh's Mymensingh district from August to December 2019. This study is a sub-study of a larger study that aimed to evaluate the effectiveness of GMP programs in improving child nutritional status [13].

We selected the Mymensingh district based on the following criteria: (A) time of implementation of the programme matching with the study timeline; (B) staff and logistics in CCs supported by NGO; (C) adequate number of CCs in subdistricts to satisfy the sample size [13], and (D) comparativeness to other Bangladeshi districts with its predominantly rural, agrarian population, along with comparable literacy rates and demographic patterns [13]. In this area, the Government of Bangladesh administered Growth Monitoring and Promotion (GMP) services via health facility-based platforms, notably community clinics (CC). These CCs, which are community-based government healthcare facilities, cater to the health, family planning, and nutrition needs of 6,000 or more households [12].

Overall, six rural sub-districts Bangladesh's Mymensingh district were selected. Three neighboring sub-districts were chosen where only the government's facility-based GMP services were available, without any NGO involvement. In addition, To understand the home-based and facility-based GMP service delivery models, three sub-districts were selected where a non-governmental organization (NGO) supported the government's facility-based GMP services with home-based initiatives. In Mymensingh, an NGO collaborated with the government to enhance the GMP program in select sub-districts including the ones selected for this study. The NGO provided home-based GMP services through its staff, using a tracking system for child monitoring. To boost GMP utilization, it developed counseling tools and ensured a consistent supply of GMP cards to caregivers, following standard GMP components outlined in the Introduction section of this study [14]. Concurrently, three neighboring sub-districts were chosen where only the government's facility-based GMP services were available, without any NGO involvement.

The home and facility-based areas weare matched based on some socio-demographic characteristics to avoid selection bias [13]. Initially, study-specific general information was collected on each CC and its catchment area through discussions with three groups of residents: (1) Community health care providers (CHCPs), (2) mothers/caregivers of under-two children, and (3) members of CC-associated community group (CG). Discussions focused on: (1) GMP service availability, (2) availability of other nutrition services at the CCs for women and children, (3) components of GMP provided by CHCP, and (4) sociodemographic status of people living in the CC catchment area. One point was given to a response if it contains more than or equal to 90% response rate (positive or negative), and less than 90% response got zero, totaling to a maximum of 5 points for a given CC. All the points were then summed to generate a combined score for each CC. Majority of the intervention area CCs (more than 90%) scored 3 out of 5. In the next stage, due to the similarities of scores, 30 intervention CCs were selected by computer-generated simple random sampling from the total 72 CCs in the intervention subdistricts. Similar scores were generated for each control CC. The 30 control CCs were then individually matched with the 30 intervention CCs based on the closest scores. Thus, the intervention-to-control CC ratio was 1:1.

We compared caregivers' knowledge of GMP service, levels of GMP service utilization, and barriers to GMP service utilization in both types of settings. This approach allowed for a comparative analysis of GMP service delivery in varied contexts within the Mymensingh district.

## **Selection and recruitment of study participants for the quantitative part of the study**

For the quantitative part of the study, all data of participants of the initial, larger study (3038 mother-child pairs) were included (13). We used proportionate sampling based on the size and population of the sub-district.

The study included children aged 6-23 months and their mothers/caregivers in Community clinics' catchment areas. Eligibility criteria encompassed children free from known acute/chronic illnesses, severe stunting, wasting, or underweight due to ethical obligation as the study would not provide treatment or management, not enrolled in similar programs, and whose caregivers provided consent. The youngest child in each household was designated as the index child, with twins excluded to prevent potential information biases. In case of participant absence, the next eligible household was approached during data collection.

## **Facility-based GMP service delivery**

The Government of Bangladesh (GoB) has implemented a Growth Monitoring and Promotion (GMP) program in its Community Clinics (CCs) since 2011. Each CC in the current study was meant to have three healthcare staff responsible for providing GMP to every child under five years of age, whether they visited for illness or routine check-ups. The GoB had developed a National Guideline for GMP implementation. It was responsible for training the CC staff and providing the necessary equipment and supplies. Despite consistency in GMP components as described above [14], there has been a concern about the need for more active supervision at the CCs [15].

## **Home-based GMP service delivery**

During the study period in Mymensingh, a single NGO partnered with the government to support the GMP program in certain sub-districts. The NGO provided extra support by engaging its staff for home-based GMP services. They offered GMP sessions at home for children using a tracking system. The NGO aimed to improve GMP utilization by creating counseling tools and ensuring a steady supply of GMP cards to caregivers at home. The GMP components were aligned with the previously outlined methodology [14].

## **GMP service utilization**

Operational definition: Caregivers' GMP service utilization was defined as whether they received GMP services at home or went to the facility for the service even once during the child’s lifetime during the survey.

## **Data processing and analysis**

### **Quantitative data collection**

Fourteen data collectors collected quantitative data from mothers/caregivers of children aged 6-12 months in their households, using a pretested semi-structured questionnaire (S2 File). The survey questionnaire covered topics including household socio-demographics, water sanitation and hygiene practices, knowledge and perception of GMP services, utilization of GMP services, perceived barriers in GMP service utilization, and child anthropometry.

Data collectors directly observed households' drinking water sources and toilets. Improved toilets were defined as flush toilets, ventilated improved pit latrines, traditional pit latrines with a slab, or composting toilets; improved drinking water sources were classified as water from tubewells [16]. The data collectors utilized Bangladesh government-approved GMP cards and asked caregivers about their knowledge of the cards, showing growth charts for interpretation. Caregivers' responses were recorded in close ended questionnaires. If caregivers had not utilized GMP services, they were asked for reasons via a multiple-option choice questionnaire.

Child anthropometry followed WHO standard operating procedures [17], with staff initially trained by certified gold standards at icddr,b. Measurements were taken using a Seca Infantometer for length and a Seca 727 Baby Scale for weight, ensuring daily precision through calibration and repeated readings when necessary. All measuring tools were placed on flat surfaces, and data collectors noted the readings when participants became steady on the scale. One data collector read the measurement, and another recorded it on the form.

**Statistical analysis for the quantitative part of the study**

The completed questionnaires were securely transported to icddr,b and received by data management assistants and a statistician for entry and analysis using MS Access and STATA software version 15, respectively. The study statisticians and investigators decoded caregivers' responses on GMP growth card questionnaires and determined "correct" responses, e.g., if a caregiver mentioned that the "red color zone" in the growth chart means the child is severely malnourished, it was considered "correct" by the investigators. The study team assessed caregivers' responses to GMP growth card questionnaires for accuracy. Child growth parameters were calculated based on WHO standards [18]. A wealth index was constructed from household assets using a composite score to classify socioeconomic status [19]. Households were grouped into three categories: rich, middle, and poor, reflecting relative differences in asset ownership.

Data were checked for normality using Skewness/Kurtosis tests, histogram and quantile-normal (Q-Q) plots (qnorm) (S1 File). Outliers were managed according to standard statistical/data analysis procedures. Categorical variables were summarized using proportions and frequencies, while normally distributed continuous variables were summarized using means and standard deviations. Student's t-tests compared counts with a normal distribution, and the chi-square distribution test compared qualitative variables for both home-based and facility-based GMP arms. The differences in caregivers' GMP knowledge between home-based and facility-based GMP proportions were reported using risk ratios (RR) with 95% confidence intervals (95% CI) without adjustments. Simple logistic regression models were used to determine caregivers' level of GMP service utilization and barriers to GMP service utilization.

Predictors of caregivers' GMP utilization were explored using logistic regression models only for the home-based group due to the absence of GMP service utilization among facility-based caregivers. The study examined factors influencing caregiver GMP service utilization versus non-utilization. All independent variables significantly associated with GMP utilization (dependent variable and primary outcome) in bivariate models and biologically plausible variables were included in the final multivariable models. The multivariable models were adjusted for relevant covariates: caregiver awareness of GMP or GMP card, NGO membership, and caregiver's lack of demand for GMP service. Estimated associations were described as adjusted risk ratios (ARR) with 95% confidence intervals (CIs). Results with p-values <0.05 were considered statistically significant.

### **Qualitative data collection**

Qualitative approaches were applied to enhance the quantitative survey findings. The study employed one moderator and one interviewer proficient in conducting qualitative interviews and analysis. The qualitative interviews were carried out by trained moderators and interviewers whose native language was Bangla. Interviews were conducted in Bangla and later translated into English. The Bangla version of the questionnaire was validated before the interview through translation and back-translation [13]. Open-ended questionnaires and guidelines were used for qualitative interviews, along with checklists for CC observations. These tools included questions on caregivers' GMP knowledge, perception, and practices, as well as CC-related GMP service information.

Two qualitative researchers (Ishrat Jahan and Ziaul Islam) employed a thematic analysis for all qualitative information.

**Statistical analysis for the qualitative part of the study**

Qualitative analysis included thematic descriptions, analysis, and quotations from respondents. Transcribed interviews were coded based on predetermined themes (a priori), subthemes, and emergent issues. Trustworthiness was ensured through four principles: credibility, transferability, conformability, and dependability [20]. Intercoder or synchronic reliability was utilized to gauge the agreement between independent coders of the data. An agreement was assessed during the analysis when the researchers independently coded the same interviews. Triangulation was conducted between methods and participants, validating information by triangulating data from different approaches.

## **Sample size and sampling technique for the qualitative part of the study**

All the data of the participants enrolled in the larger study (3038 mother-child pairs) were included in the quantitative analysis (13). We used proportionate sampling based on the size and population of the sub-district.

Only some relevant participants (n=36) were interviewed for the qualitative part. The qualitative participants were selected in a purposive manner from the program areas to supplement the quantitative findings. However, we did not collect their detailed socio-demographic characteristics to make a comparison. The number of qualitative interviews was determined through an iterative process, where data from each round of interviews were analyzed to identify emerging themes. Interviews continued until information saturation was reached—that is, when no new insights were gained, indicating that key themes had been thoroughly explored.

## **Ethical considerations**

The Institutional Review Board (IRB) of icddr,b approved the study (PR-17123, version 1.02, dated 06 March 2018). Written informed consent with full disclosure about the study was taken from the participants before all interviews. The privacy of the respondents, their anonymity, and the confidentiality of data/information were strictly maintained. Only the principal investigators of this study had access to information on personal identification and other sensitive information that the respondents provided.

# **Results**

## **Sociodemographic characteristics of the study participants**

A total of 3038 mother-child dyads were enrolled in this study between August and December 2019, evenly split between home-based and facility-based service areas (Fig 1). We excluded children during household screening which is about 16% (n=291) in intervention area and 7% (n=122) in control area. Sixteen qualitative in-depth interviews (IDIs) were conducted with community health care providers (CHCPs )and 20 with mothers of enrolled children. In addition, daylong indirect observations were conducted at 23 CCs.

**Fig 1. Participant flow chart**

Children's average age was 9.8 months, with a 1:1 male-to-female ratio in both groups (home-based vs. facility-based: 51.9% vs. 50.0%). Children's length-for-age Z-score (LAZ) and weight-for-age Z score (WAZ) was comparable among the groups. Children from home-based GMP area had significantly lower weight-for-length/height Z-score (WHZ) compared to facility-based area children (mean WHZ: -0.48 versus -0.4, p=0.04). Mother’s age (home-based vs. facility-based: 26.1 vs 25 years) and father’s years of education (home-based vs. facility-based: 7.4 vs 6.9 years) were significantly higher in home-based GMP area compared to facility-based GMP area (p=0.01 and 0.002 respectively) (Table 1). Approximately 98% of household members in both areas identified as Muslim, with 94% and 92% having improved toilets in home-based and facility-based GMP service areas, respectively (p=0.016) (Table 1). More (44%) caregivers in home-based GMP areas were from poor families compared to facility-based GMP areas (38%) (p=0.002) (Table 1). The socio-demographic characteristics of the sub-districts in 2 GMP areas were mostly comparable (S1 file).

**Table 1.** **Caregiver's sociodemographic characteristics across home-based (**N = 1,519) **and facility-based (**N = 1,519) **GMPs**

| **Characteristic** | | **Home-based GMP** | **Facility-based GMP** | **p-value** |
| --- | --- | --- | --- | --- |
| Child age in months, mean (SD) | | 9.9 (1.7) | 9.7 (1.8) | 0.065 |
| Child's weight-for-age Z-score (WAZ), mean (SD) | | -1.1 (1.1) | -1.1 (1.1) | 0.555 |
| Child's length-for-age Z-score (LAZ), mean (SD) | | -1.3 (1.1) | -1.4 (1.1) | 0.190 |
| Child's weight-for-length Z-score (WHZ), mean (SD) | | -0.48 (0.03) | -0.40 (0.03) | 0.043 |
| Proportion of boys, n (%) | | 760 (50.0) | 789 (51.9) | 0.293 |
| Mother's age in years, mean (SD) | | 26.1 (5.8) | 25.6 (5.9) | 0.011 |
| Mother's education in years, mean (SD) | | 6.1 (3.6) | 6.1 (3.6) | 0.717 |
| Mother's occupation, n (%) | |  |  |  |
| Housewife/homemakers | | 1,474 (97.0) | 1,487 (97.9) | 0.133 |
| Fathers' education in years, mean (SD) | | 7.4 (4.5) | 6.9 (3.4) | 0.002 |
| Religion, n (%) | |  |  |  |
|  | Islam | 1,494 (98.4) | 1,478 (97.3) | 0.046 |
|  | Hinduism/Buddhism/Christian | 25 (1.6) | 41 (2.7) |  |
| Improved toilet facility, n (%) | | 1,402 (92.3) | 1,435 (94.5) | 0.016 |
| Improved source of drinking water, n (%) | | 1,514 (99.7) | 1,515 (99.7) | 0.739 |
| Wealth index, n (%) | |  |  |  |
|  | Poor | 667 (44.0) | 577 (38.0) | 0.002 |
|  | Middle | 430 (28.3) | 461 (30.3) |  |
|  | Rich | 420 (27.7) | 481 (31.7) |  |

## **Caregiver's knowledge and perception of GMP service**

Caregivers from home-based GMP areas had higher GMP knowledge levels compared to those in facility-based areas. In home-based GMP areas, a higher proportion of caregivers (67%) had heard about GMP or GMP cards compared to facility-based GMP areas (0.4%) (RR: 169, 95% CI: 76, 376; respectively) (Table 2). However, only 17% of caregivers in home-based GMP areas correctly understood the purpose of the growth chart and explained the meaning of different colors compared to less than 1% in facility-based GMP areas (RR: 127.5 and 122.5, , and 31, 492, respectively). A total of 2,791 (91.8%) out of 3,038 responses from mothers were incorrect; Among them intervention, n=1,274 (83.8%) and control, n=1,517 (99.8%) responses were incorrect. More than half of the caregivers (51%) in home-based GMP service areas mentioned GMP service as “beneficial”, while none from facility-based regions did. The meaning of the word “beneficial” (“*valo/upokari”* in Bangla) was not explained to the respondent, but it was left for them to judge.

**Table 2:** **Caregiver's knowledge of GMP service in facility and home-based GMP program**

| **Indicators** | **Home-based GMP**  N = 1,519 | **Facility-based GMP**  N = 1,519 | **RR (95% CI)** | **p-value** |
| --- | --- | --- | --- | --- |
| Caregivers heard about GMP or GMP card | 1,014 (66.8) | 6 (0.4) | 169 (76, 375.9) | <0.001 |
| Caregivers know the purpose of the growth chart | 255 (16.8) | 2 (0.1) | 127.5 (31.8, 511.6) | <0.001 |
| Caregivers can correctly explain the colors in the GMP card growth chart | 245 (16.1) | 02 (0.13) | 122.5 (30.5, 491.6) | <0.001 |
| Caregivers mentioned GMP service as "beneficial" | 777 (51.2) | 0 (0) | - | - |

Growth monitoring and promotion, GMP; confidence interval, CI; Risk Ratio, RR.

## **Caregiver's level of GMP service utilization**

GMP service utilization was significantly higher in home-based areas compared to facility-based areas. In home-based GMP service areas, 61% of caregivers had received GMP cards (Table 3), while none had done so in facility-based GMP areas. Significantly more children underwent weight measurement, length measurement, and their caregivers received growth monitoring-specific counseling in home-based GMP service compared to facility-based GMP service (81% vs. 21%; 3.4% vs. 0.6%; and 55% vs. 06%, RR: 4, 5.7 and 9.5, respectively). However, only 30 out of 1519 children and their caregivers received all three major components of GMP services (child weight, length measurement, and GM-specific counseling) in a single GMP visit at home-based GMP services (Table 3). Children and their caregivers in facility-based areas never received all three components together. Overall, 40% of caregivers from home-based GMP service areas utilized GMP service compared to 0% in facility-based GMP service (Table 3).

**Table 3.** **Levels of GMP service utilization among individuals in facility (**N = 1,519) **and home-based (**N = 1,519) **GMP programs(**

| **Indicators** | **Home-based GMP N (%)** | **Facility-based GMP N (%)** | **RR (95% CI)** | **p-value** |
| --- | --- | --- | --- | --- |
| Caregivers received a GMP card | 926 (60.9) | 0 (0) | - | - |
| The child was weighed at least once | 1231 (81.0) | 311 (20.5) | 4.0 (3.6, 4.4) | <0.001 |
| The child length was measured at least once | 51 (3.36) | 09 (0.59) | 5.7 (2.8, 11.5) | <0.001 |
| Caregivers got child growth monitoring-specific counseling | 837 (55.2) | 88 (5.4) | 9.5 (7.7, 11.7) | <0.001 |
| The child received all major components of GMP (length, weight, and growth monitoring-specific counseling) altogether | 30 (1.97) | 0 (0) | - | - |
| Caregivers utilized GMP services | 603 (39.7) | 0 (0) | - | - |

Growth monitoring and promotion, GMP; confidence interval, CI; Risk Ratio, RR.

## **Barriers to caregivers' access and utilization of GMP services**

Four major barriers were identified as reasons for caregivers' underutilization of GMP services. Among these, caregivers' lack of knowledge about the availability of GMP services at the nearest health facility was a significant barrier in both areas (Table 4). Caregivers from facility-based GMP areas had significantly less knowledge about GMP service availability compared to those in home-based service areas (52% vs. 61%, respectively; RR: 0.9, 95% CI: 0.8, 0.9). Additionally, caregivers' demand for GMP service was significantly lower in facility-based areas compared to home-based GMP areas (55% vs. 44%, respectively; RR: 0.7). More caregivers from home-based GMP service areas reported closure of health facilities/absence of service providers (1%) and being preoccupied with household work (4%) as significant barriers to accessing and utilizing GMP services, compared to facility-based GMP service areas (0.2% and 0.3%, respectively) (Table 4).

In-depth interviews (IDIs) with mothers/caregivers provided insight into the obstacles to accessing GMP services in both settings. Mothers/caregivers in the home-based GMP area mainly relied on NGO staff for GMP service delivery at home, bypassing visits to CCs for GMP services. In contrast, mothers in facility-based GMP areas reported that government community health workers never visited their communities or homes to inform them about GMP services at CCs or the nearest health facilities. Caregivers from facility-based GMP areas never received GMP cards because of lack of supply of GMP cards in CCs from the government. Interviews with government stakeholders revealed the lack of allocation of government funds to print and supply GMP cards to the CCs during the study period. The NGO area caregivers got GMP cards as the NGO printed out and distributed government approved GMP cards using their own funds. Caregivers from both areas expressed unawareness of GMP services available at CCs or nearby health facilities. Additionally, caregivers mentioned a preference for accessing health services/programs that offered incentives or immediate benefits, such as vaccines, cash, food, vitamins, or supplements. As neither the home-based nor the facility-based GMP program provided such incentives, mothers/caregivers lacked interest in utilizing these services.

**Table 4:** **Barriers to GMP service utilization among individuals in the facility (**N = 1,519) **and home-based (**N = 1,519) **GMP^a^ programs**

| **Indicators** | **Home-based GMP N (%)** | **Facility-based GMP N (%)** | **RR (95% CI)** | **p-value** |
| --- | --- | --- | --- | --- |
| The caregiver was preoccupied with household chores and did not go for the GMP service | 56 (3.7) | 5 (0.3) | 11.2 (4.5, 27.9) | <0.001 |
| The health facility was closed/no healthcare provider at the facility | 14 (0.9) | 3 (0.2) | 4.6 (1.3, 16.2) | 0.015 |
| Caregivers' lack of interest in GMP services at an individual level | 668 (44.0) | 834 (54.9) | 0.7 (0.6, 0.9) | 0.006 |
| Lack of knowledge of GMP service availability at the nearest health facility | 798 (52.5) | 919 (60.5) | 0.9 (0.8, 0.9) | <0.001 |

Growth monitoring and promotion, GMP; confidence interval, CI; Risk Ratio, RR.

^a^Multiple responses by caregivers.

## **Predictors of caregivers' GMP service utilization**

Utilization of GMP services was positively associated with caregivers' knowledge of GMP or GMP card (Adjusted risk ratio, ARR: 37.4, 95% confidence interval) and membership of an NGO/association/health program (ARR: 1.3) (Table 5). A key barrier to GMP service utilization was caregivers' low interest in the service (ARR: 0.7).

**Table 5. Predictors of GMP service utilization among caregivers at the home-based GMP programs**

| **Characteristic^a^** | **Characteristic category** | **RR (95% CI)** | **p-value** | **ARR (95% CI)** | **p-value** |
| --- | --- | --- | --- | --- | --- |
| Caregiver having heard about GMP or GMP card | No^1^ | - | - | - | - |
|  | Yes | 42.4 (20.3, 88.6) | <0.001 | 37.4 (17.8, 78.5) | <0.001 |
| Caregiver being a member of an association/NGO/health program | No^1^ | - | - | - | - |
|  | Yes | 2.6 (2.1, 3.2) | <0.001 | 1.3 (1.1, 1.5) | 0.001 |
| Caregiver's low interest in GMP services | No^1^ | - | - | - | - |
|  | Yes | 0.5 (0.4, 0.7) | <0.001 | 0.7 (0.5, 0.9) | 0.001 |

RR: Risk ratio, CI: Confidence interval, ARR: Adjusted risk ratio, Growth monitoring and promotion, GMP; Non-governmental organization, NGO; and 1 = reference category

^a^The Model included caregivers who heard about GMP or GMP cards, were members of an NGO, and lacked interest in GMP service.

# **Discussion**

The study aimed to assess caregivers' knowledge, perception, utilization, and barriers to accessing GMP services in rural Bangladesh, comparing home-based and facility-based settings. In a sample of 1519 families from 3 rural subdistricts, caregivers from home-based GMP areas had higher GMP knowledge levels compared to facility-based GMP areas. Sixty-one percent of caregivers received GMP cards at home-based GMP, whereas none did in facility-based regions due to a lack of supply. This explains why only 0.4% of caregivers would have heard of GMP cards in facility based GMP areas. However, we could not compare these results with government or other research data, since our study is the first to report this and there is no such information available. More children were measured for weight and length at home-based services, and their caregivers received growth monitoring-specific counseling compared to facilities. GMP service utilization was higher among caregivers at home compared to facility-based GMP services (40% vs. 0%, respectively). The stark contrast in GMP service utilization, with home-based services at 40% and facility-based services at 0%, underscores critical barriers in accessing and delivering care. While home-based services perform better, their utilization remains lower than expected due to irregular or insufficient visits by health workers, limited caregiver awareness of GMP’s importance, and competing priorities or time constraints [21]. In contrast, the zero utilization of facility-based GMP highlights additional challenges such as limited accessibility to health facilities, lack of awareness or trust in facility-based services, and caregivers’ preference for more convenient or culturally appropriate home-based interventions [22]. Moreover, the absence of facility-based utilization may reflect systemic gaps in infrastructure, staffing, and health system outreach efforts [6].

The validity and reliability of the study findings could theoretically have been reduced by several factors, including the sole presence of one NGO offering home-based GMP services, the longstanding integration of this NGO within the community affecting caregivers' healthcare choices, the accessibility of home-based GMP services, exclusion of children with known chronic illness, severely malnourished children and twins, and the relatively brief duration of the study. Nonetheless, the presence of similar sociodemographic characteristics among participants helped alleviate potential biases arising from the NGO's sole presence. Despite the potential impact of the NGO and the accessibility of home-based GMP services, the low utilization rates in the home-based GMP area indicate a limited effect. Our study included only apparently healthy, disease-free children, which might introduce a selection bias and limit the generalizability of the findings to children with underlying health conditions. We did not collect information from excluded children and thus could not assess the impact on study findings and GMP service utilization matrix. We excluded twins during screening to reduce potential information bias. However, despite the study's short duration, its findings offer valuable insights into GMP utilization trends in rural settings. These findings underscore that caregiver of infants in rural Bangladesh significantly engaged with home-based GMP services, while facility-based services were not used at all. The validity and reliability of these study findings are upheld through rigorous observation, robust study design, and mitigation of potential biases.

Our finding on low service utilization despite better knowledge of GMP is consistent with other LMIC settings [6, 23, 24]. No other study has ever documented no GMP use in facilities, which is unique to this setting and contrasts with the existing LMIC GMP program findings (5, 22, 23, 26). This contrasting finding may be due to the influence of differences in cultural beliefs, social norms, and contextual factors [22]. This study is first to report on the absence of perceived benefit in facility-based sub-districts, which is unique as there is no government or research data available. Caregivers from both home- and facility-based GMP settings prefer curative services over preventive services like GMP, which is also expected in LMIC settings [6, 25, 26]. They only take their child to health facilities when sick, either for treatment or to obtain free medicines [6, 27]. Additionally, caregivers prefer incentives or immediate benefits, such as vaccines or cash, highlighting the importance of addressing these preferences to increase the utilization of GMP services, which several other studies also reported [5-7, 25, 26].

Caregivers' knowledge, perceptions, and barriers differ between home-based and facility-based GMP areas. Home-based services are better utilized due to NGO presence [11, 28, 29]. However, gaps exist in understanding growth charts, requiring clearer explanations from health workers [5, 30]. Caregivers’ awareness of GMP services is limited due to their reliance on NGOs in home-based areas, where government outreach is minimal. In addition, there are infrequent visits by government staff to health facilities which further hinders the utilization by caregivers of young children. This gap in engagement and service delivery reduces caregivers' understanding and access to GMP services. The insufficient supply of GMP cards in government facilities significantly contributes to their non-utilization and highlights a critical structural issue within the health system [26, 31, 32]. This shortfall stems from disrupted supply chains, limited funding, or inadequate prioritization of GMP services, ultimately hindering caregivers’ ability to track and engage with child growth monitoring [32]. Barriers like health facility closures and household responsibilities also explain low utilization rates [6]. To address this, systemic reforms are needed to ensure a reliable supply of GMP cards, as they are essential tools for promoting effective service delivery and improving child health outcomes.

The findings emphasize the necessity for GMP programs tailored to caregivers' needs [4], highlighting differences between home and facility areas. Clear communication strategies are needed to address gaps in understanding growth charts [30]. Improvement is required to ensure caregivers receive all GMP components during home visits. Barriers like reliance on NGOs and limited government visits hinder service awareness. Overcoming these barriers, such as providing GMP cards and improving caregiver understanding, is crucial for enhancing program coverage [27, 32]. Strengthening partnerships with NGOs and community organizations can promote caregivers' GMP service utilization [33, 34]. To improve GMP utilization, specific strategies for integrating NGO initiatives into the public health system should focus on strengthening coordination and resource sharing. This includes joint training of staff, aligning service delivery goals, and establishing referral and follow-up mechanisms between home-based and facility-based services [33, 34]. Such integration can bridge service gaps, enhance caregiver awareness, and improve access to GMP services.

The study highlights the critical role of accessibility and convenience in influencing GMP utilization patterns. Home-based GMP services, despite their availability, exhibited low utilization rates, suggesting that accessibility alone may not drive engagement. Factors such as awareness, perceived benefits, and trust in service providers likely played a role in shaping caregivers' decisions. Additionally, the study acknowledges that the two populations (facility based-GMP and home based-GMP) had some baseline differences beyond just the type of GMP received. These initial differences may have influenced the outcomes observed, potentially confounding the effects attributed solely to GMP type. While our analysis aimed to account for these variations, the findings should be interpreted with caution, considering that factors could have contributed to the observed differences between groups. Future research should explore these factors further to provide a more comprehensive understanding of GMP utilization dynamics.

This study has four features that limit the conclusions that one can draw from the findings. Firstly, it was conducted in only a few subdistricts of rural Bangladesh, excluding urban areas, potentially compromising the representation of the country's overall GMP utilization scenario. Secondly, its cross-sectional design inhibits the establishment of causal relationships between caregivers' GMP utilization and associated factors. Thirdly, the absence of longitudinal data prevents the assessment of long-term impact. Fourth, the absence of facility-based GMP utilization during the study period precludes the determination of predictors influencing caregivers' GMP service utilization.

# **Conclusion**

This study contrasts home-based and facility-based GMP services in rural Bangladesh, favoring home-based options for caregivers with infants 6-23 months of age. The involvement of local NGOs underscores the importance of tailored, accessible approaches to meet caregivers' needs. Policymakers should prioritize integrating home-based GMP services within existing healthcare systems to optimize child health outcomes.

# **Acknowledgments**

The study co-authors acknowledged the use of ChatGPT 3.5, developed by OpenAI, to edit the English language. The study team also acknowledges the role of two qualitative researchers, Ms. Ishrat Jahan and Dr. Ziaul Islam, for their contribution to qualitative data collection and analysis.

**Competing interest statement:** None declared

**Disclaimer:** The funders had no role in the study design, data collection, analysis, or interpretation. The research was conducted independently of the funders, and the views expressed in this protocol are those of the authors.

**Competing interests:** None declared.

# **Reference**

1.	Ashworth A, Shrimpton R, Jamil K. Growth monitoring and promotion: review of evidence of impact. Matern Child Nutr. 2008;4 Suppl 1(Suppl 1):86-117. doi: 10.1111/j.1740-8709.2007.00125.x. PubMed PMID: 18289158; PubMed Central PMCID: PMCPMC6860476.

2.	Hossain M, Choudhury N, Adib Binte Abdullah K, Mondal P, Jackson AA, Walson J, et al. Evidence-based approaches to childhood stunting in low and middle income countries: a systematic review. Arch Dis Child. 2017;102(10):903-9. Epub 20170503. doi: 10.1136/archdischild-2016-311050. PubMed PMID: 28468870; PubMed Central PMCID: PMCPMC5739821.

3.	Singh A, Torres KA, Maharjan N, Shrestha J, Agbozo F, Abubakari A, et al. Learning from health system actor and caregiver experiences in Ghana and Nepal to strengthen growth monitoring and promotion. PLoS One. 2023;18(3):e0282807. Epub 20230309. doi: 10.1371/journal.pone.0282807. PubMed PMID: 36893119; PubMed Central PMCID: PMCPMC9997959.

4.	Bégin F, Elder L, Griffiths M, Holschneider S, Piwoz E, Ruel-Bergeron J, et al. Promoting Child Growth and Development in the Sustainable Development Goals Era: Is It Time for New Thinking? J Nutr. 2020;150(2):192-4. doi: 10.1093/jn/nxz244. PubMed PMID: 31599947.

5.	Feleke FW, Adole AA, Bezabih AM. Utilization of growth monitoring and promotion services and associated factors among under two years of age children in Southern Ethiopia. PLoS One. 2017;12(5):e0177502. Epub 20170516. doi: 10.1371/journal.pone.0177502. PubMed PMID: 28510596; PubMed Central PMCID: PMCPMC5433735.

6.	Pollifrone MM, Cunningham K, Pandey Rana P, Philbin MM, Manandhar S, Lamsal KP, et al. Barriers and facilitators to growth monitoring and promotion in Nepal: Household, health worker and female community health volunteer perceptions. Matern Child Nutr. 2020;16(4):e12999. Epub 20200712. doi: 10.1111/mcn.12999. PubMed PMID: 32657015; PubMed Central PMCID: PMCPMC7507439.

7.	Bilal SM, Moser A, Blanco R, Spigt M, Dinant GJ. Practices and challenges of growth monitoring and promotion in Ethiopia: a qualitative study. J Health Popul Nutr. 2014;32(3):441-51. PubMed PMID: 25395907; PubMed Central PMCID: PMCPMC4221450.

8.	Charlton KE, Kawana BM, Hendricks MK. An assessment of the effectiveness of growth monitoring and promotion practices in the Lusaka district of Zambia. Nutrition. 2009;25(10):1035-46. Epub 20090805. doi: 10.1016/j.nut.2009.03.008. PubMed PMID: 19660911.

9.	Hossain SMM, Duffield A, Taylor A. An evaluation of the impact of a US$60 million nutrition programme in Bangladesh. Health Policy and Planning. 2005;20(1):35-40.

10.	Gwatkin DR, Bhuiya A, Victora CG. Making health systems more equitable. Lancet. 2004;364(9441):1273-80. doi: 10.1016/s0140-6736(04)17145-6. PubMed PMID: 15464189.

11.	Fink G, Levenson R, Tembo S, Rockers PC. Home- and community-based growth monitoring to reduce early life growth faltering: an open-label, cluster-randomized controlled trial. Am J Clin Nutr. 2017;106(4):1070-7. Epub 20170823. doi: 10.3945/ajcn.117.157545. PubMed PMID: 28835364; PubMed Central PMCID: PMCPMC5611784.

12.	Riaz BK, Ali L, Ahmad SA, Islam MZ, Ahmed KR, Hossain S. Community clinics in Bangladesh: a unique example of public-private partnership. Heliyon. 2020;6(5).

13.	Hossain M, Ferdous TE, Islam Z, Billah SM, Bulbul MMI, Rahman MM, et al. Protocol for a quasiexperimental study testing the effectiveness of strengthening growth monitoring and promotion in community clinics for improving the nutritional status of under-two children in rural Bangladesh. BMJ Open. 2019;9(10):e032458. Epub 20191010. doi: 10.1136/bmjopen-2019-032458. PubMed PMID: 31601604; PubMed Central PMCID: PMCPMC6797324.

14.	Panpanich R, Garner P. Growth monitoring in children. Cochrane Database Syst Rev. 2000;(2):Cd001443. doi: 10.1002/14651858.Cd001443. PubMed PMID: 10796794.

15.	Ministry of Health and Family Welfare DGoHS, Community Based Health Care programme, World Health Organization (WHO) Bangladesh. Independent evaluation of community-based health services in Bangladesh. Dhaka: Community Based Health Care (CBHC) Directorate General of Health Services (DGHS) Health Services Division, Ministry of Health and Family Welfare Government of the People’s Republic of Bangladesh and World Health Organization (WHO) Bangladesh, 2019.

16.	National Institute of Population ResearchTraining - NIPORT/Bangladesh, Mitra and Associates, ICF International. Bangladesh Demographic and Health Survey 2014. Dhaka, Bangladesh: NIPORT, Mitra and Associates, and ICF International, 2016.

17.	de Onis M, Onyango A, Borghi E, Siyam A, Blössner M, Lutter C. Worldwide implementation of the WHO Child Growth Standards. Public Health Nutr. 2012;15(9):1603-10. Epub 20120412. doi: 10.1017/s136898001200105x. PubMed PMID: 22717390.

18.	World Health Organization (WHO). WHO child growth standards : length/height-for-age, weight-for-age, weight-for-length, weight-forheight and body mass index-for-age : methods and development. France: WHO; 2006.

19.	Filmer D, Pritchett LH. Estimating wealth effects without expenditure data—or tears: an application to educational enrollments in states of India. Demography. 2001;38(1):115-32.

20.	Ahmed SK. The pillars of trustworthiness in qualitative research. Journal of Medicine, Surgery, and Public Health. 2024;2:100051. doi: https://doi.org/10.1016/j.glmedi.2024.100051.

21.	Faber M, Phungula MA, Kvalsvig JD, Benadé AJ. Acceptability of community-based growth monitoring in a rural village in South Africa. Food Nutr Bull. 2003;24(4):350-9. doi: 10.1177/156482650302400405. PubMed PMID: 14870622.

22.	Teklemuz N, Sisay M, Baffa LD, Mengistu B, Atenafu A. Utilization of growth monitoring and promotion services among children younger than 2 years in West Armachiho district, Northwest Ethiopia. Frontiers in Public Health. 2023;11. doi: 10.3389/fpubh.2023.1179720.

23.	Newton-Lewis TA, Bahety G. Evaluating the effectiveness of Community Health Worker home visits on infant health: A quasi-experimental evaluation of Home Based Newborn Care Plus in India. J Glob Health. 2021;11:04060. Epub 20211009. doi: 10.7189/jogh.11.04060. PubMed PMID: 34737860; PubMed Central PMCID: PMCPMC8542379.

24.	Sudfeld CR, Bliznashka L, Ashery G, Yousafzai AK, Masanja H. Effect of a home-based health, nutrition and responsive stimulation intervention and conditional cash transfers on child development and growth: a cluster-randomised controlled trial in Tanzania. BMJ Glob Health. 2021;6(4). doi: 10.1136/bmjgh-2021-005086. PubMed PMID: 33906847; PubMed Central PMCID: PMCPMC8088247.

25.	Agbozo F, Colecraft E, Jahn A, Guetterman T. Understanding why child welfare clinic attendance and growth of children in the nutrition surveillance programme is below target: lessons learnt from a mixed methods study in Ghana. BMC Nurs. 2018;17:25. Epub 20180619. doi: 10.1186/s12912-018-0294-y. PubMed PMID: 29950927; PubMed Central PMCID: PMCPMC6009038.

26.	Tekle M, Tariku B, Alagaw A, Zerihun E, Bekele HW. Exploring Reasons for Low Attendance of Mothers to Growth Monitoring and Promotion Program at Loka Abaya District, Southern Ethiopia: Exploratory Qualitative Study. J Nutr Metab. 2019;2019:3510649. Epub 20190224. doi: 10.1155/2019/3510649. PubMed PMID: 30918722; PubMed Central PMCID: PMCPMC6409037.

27.	Nguyen PH, Khương LQ, Pramanik P, Billah SM, Menon P, Piwoz E, et al. Effective coverage of nutrition interventions across the continuum of care in Bangladesh: insights from nationwide cross-sectional household and health facility surveys. BMJ Open. 2021;11(1):e040109. Epub 20210120. doi: 10.1136/bmjopen-2020-040109. PubMed PMID: 33472778; PubMed Central PMCID: PMCPMC7818835.

28.	Shonchoy AS, Akram AA, Khan M, Khalid H, Mazhar S, Khan A, et al. A Community Health Worker–Based Intervention on Anthropometric Outcomes of Children Aged 3 to 21 Months in Urban Pakistan, 2019–2021. American Journal of Public Health. 2023;113(1):105-14. doi: 10.2105/ajph.2022.307111. PubMed PMID: 36516383.

29.	Nisbett N, Davis P, Yosef S, Akhtar N. Bangladesh’s story of change in nutrition: Strong improvements in basic and underlying determinants with an unfinished agenda for direct community level support. Global Food Security. 2017;13:21-9. doi: https://doi.org/10.1016/j.gfs.2017.01.005.

30.	Roberfroid D, Pelto GH, Kolsteren P. Plot and see! Maternal comprehension of growth charts worldwide. Tropical Medicine & International Health. 2007;12(9):1074-86. doi: https://doi.org/10.1111/j.1365-3156.2007.01890.x.

31.	Tesfa M, Gonete KA, Chane Y, Yohannes S. Growth Monitoring Practice and Associated Factors Among Health Professionals at Public Health Facilities of Bahir Dar Health Centers, Northwest Ethiopia, 2021. Pediatric Health Med Ther. 2022;13:195-215. Epub 20220520. doi: 10.2147/phmt.S355214. PubMed PMID: 35620235; PubMed Central PMCID: PMCPMC9128747.

32.	Sulley I, Abizari AR, Ali Z, Peprah W, Yakubu HG, Forfoe WW, et al. Growth monitoring and promotion practices among health workers may be suboptimal despite high knowledge scores. BMC Health Serv Res. 2019;19(1):267. Epub 20190429. doi: 10.1186/s12913-019-4103-4. PubMed PMID: 31035985; PubMed Central PMCID: PMCPMC6489312.

33.	El Arifeen S, Christou A, Reichenbach L, Osman FA, Azad K, Islam KS, et al. Community-based approaches and partnerships: innovations in health-service delivery in Bangladesh. The Lancet. 2013;382(9909):2012-26.

34.	Marek T, Diallo I, Ndiaye B, Rakotosalama J. Successful contracting of prevention services: fighting malnutrition in Senegal and Madagascar. Health Policy Plan. 1999;14(4):382-9. doi: 10.1093/heapol/14.4.382. PubMed PMID: 10787654.

# Supporting information

S1 File. Details on statistical analysis

S2 File. Study questionnaire in English

S3 File. Study data set
