# Supplementary material for: Growth monitoring and promotion program services utilization patterns between home-based and facility-based delivery methods: A comparative analysis
Source: PLoS One. 2025 Jun 5;20(6):e0324918. doi: 10.1371/journal.pone.0324918 (PMC12140421; doi:10.1371/journal.pone.0324918)
Supplement: Attachment — Submitted filename: Response to the 2nd round review comments.docx [file pone.0324918.s012.docx]

17 April 2025

Zeheng Wang
Academic Editor
PLOS ONE

California, USA

RE: PONE-D-24-20319R1 - [EMID:5fd73ab87d3e57fd]: Growth monitoring and promotion program services utilization patterns between home-based and facility-based delivery methods: a comparative analysis

Dear Academic Editor,

We thank you for your response to our manuscript. Please find attached a revised version, in response to your invitation to resubmit the paper to PLOS ONE if we felt that we could satisfactorily address the concerns of the reviewers. We have substantially modified the manuscript, considering the comments from the four reviewers.

Our response to the comments is presented using the following structure: The original editorial / referee comment is copied into this letter in italics. Our response to the comment is written below in normal text format. In the case of reviewer SV- we addressed her comments inside the manuscript and highlighted them as there were more than 100 comments and accepted the suggested edits. In case a revision to the manuscript text has been made, we give the appropriate page number(s). Changes in the text have been indicated using yellow text highlighting in the manuscript and copied into this letter in the same format. The highlighted text file has been uploaded into your electronic system as a “Revised Manuscript with Track Changes and highlighted”.

**Responses to the comments made by reviewer # 5:**
*The manuscript is well-written but contains redundancies in some sections. The introduction could be more concise, avoiding repetitive statements about the importance of GMP. Some statistical results could be integrated into the discussion to provide stronger interpretation of findings and improve the depth of the discussion.*

Our response: We appreciate your kind observation. We now have revised the introduction, reads as follows (lines 60-71):

“Growth Monitoring and Promotion (GMP) is a key part of child health programs that involve regularly measuring a child’s growth to detect early signs of growth faltering and support timely interventions. The GMP program is designed to include regular check-ups for child growth and happens every three months at health facilities. In an ideal GMP program, Health workers are advised to counsel mothers and caregivers on their children's nutrition, measure the weight, length, or mid-upper arm circumference (MUAC) of all under-2-year-old children, and plot the results on a growth monitoring chart in the GMP card. If a health worker identifies a child as acutely ill or not growing well during GMP, he/she should refer the child to the nearest medical/treatment center [1]*.* This approach aims to foster communication and interaction with caregivers, promoting optimal child development [2]. UNICEF’s introduction of the promotion component in the mid-1980s strengthened GMP, making it a key part of nutrition programs for managing child malnutrition in low- and middle-income countries [3].”

The discussion section already contains results (lines 485-492):

“Sixty-one percent of caregivers received GMP cards at home-based GMP, whereas none did in facility-based regions due to a lack of supply. This explains why only 0.4% of caregivers have heard of GMP cards in facility-based GMP areas. ………More children were measured for weight and length at home-based services, and their caregivers received growth monitoring-specific counseling compared to facilities (81% vs. 21%; 3.4% vs. 0.6%; and 55% vs. 05%, respectively). GMP service utilization was higher among caregivers at home compared to facility-based GMP services (40% vs. 0%, respectively).”

*While the discussion section provides good insights, it could be expanded to:
o Compare findings with other countries that have implemented home-based GMP.*

Our response: Edited as suggested, reads as follows (lines 521-537):

“Our finding on low service utilization despite better knowledge of GMP in home-based settings is consistent with other LMIC settings [10, 16, 25, 26, 29]. Few studies have documented such findings, which is unique to this setting and contrast with the existing LMIC GMP program findings [9, 28, 29].This contrasting finding may be due to the influence of differences in cultural beliefs, social norms, and contextual factors [26]. First, caregivers may perceive home-based GMP services as more accessible and convenient, particularly in rural settings where transportation barriers, long distances, or opportunity costs (e.g., loss of income or time away from household responsibilities) hinder facility visits [10, 30]. Second, the longstanding presence of the NGO offering home-based services may have shaped caregiver preferences and trust, leading to reduced engagement with facility-based alternatives. Third, social norms and community practices—such as relying on community health workers rather than formal health facilities—may have further contributed to the non-use of facility-based GMP. Finally, weak accountability and inadequate supervision of GMP delivery at community clinics may result in poor service quality, discouraging caregivers from seeking these services [20].Together, these contextual and structural factors likely contributed to the divergence between caregiver knowledge and service utilization, underscoring the need to strengthen facility-based GMP systems while understanding and addressing local behavioral drivers.”

*o Elaborate on potential policy changes to integrate NGO-driven GMP services into government programs*

Our response: Edited as suggested, reads as follows [lines 593-606]:

“Strengthening partnerships between the government, NGOs, and community organizations is key to improving home-based GMP service utilization. Integrating NGO-led initiatives into the public health system could enhance the continuity and reach of services. This could involve joint training of health workers, shared protocols, and coordinated referral systems between home- and facility-based services. The success of NGO-supported, home-based GMP highlights the potential of community-based approaches, but scaling these models requires embedding them within national health systems. A hybrid approach, combining NGO innovations with government structures, could be effective, with shared workforce development and unified monitoring tools. To assess the feasibility of this hybrid government-NGO model, cost-effectiveness analyses should compare home-based and facility-based GMP delivery costs and outcomes. Evidence showed home-based GMP can become cost-effective when integrated with existing health platforms [14, 39, 40]. Though start-up costs may be high, integration with existing services and use of digital tools can improve cost-effectiveness and scalability over time [40].”

*o Address the scalability and sustainability of home-based GMP programs.*

Our response: Edited as suggested, reads as follows [lines 578-592]:

“Home-based GMP services, despite their availability, exhibited low utilization rates, suggesting that accessibility alone may not drive engagement. Factors such as awareness, perceived benefits, and trust in service providers played a role in shaping caregivers' decisions. The observed differences in service utilization between home- and facility-based areas suggest the importance of tailoring program strategies to address setting-specific barriers and facilitators. For example, improved communication strategies are essential to enhance caregivers’ understanding of growth charts—particularly explaining what the charts signify, how to interpret the colored zones, and what actions should be taken based on a child’s growth trajectory [34, 39]. Efforts should also ensure that all GMP components—such as weight monitoring, chart interpretation, and counseling—are consistently delivered during home visits. This could include structured home-visit checklists, routine supervision, and refresher training for frontline health workers. Barriers such as over-reliance on NGOs and limited government outreach reduce caregivers' awareness and access to GMP services. Addressing these challenges may require ensuring a consistent supply of GMP cards through improved supply chain coordination and introducing community-based demonstrations.”

*o Discuss the long-term health outcomes of children engaged in these services*

Our response: Edited as suggested, reads as follows [lines 618-621]:

“While this study did not assess long-term outcomes, prior evidence from LMICs suggests that consistent GMP engagement—particularly when combined with responsive counseling—can lead to earlier identification of growth faltering, improved caregiver feeding practices, and ultimately better child nutrition, development outcomes and productivity [11, 13, 40]. Longitudinal research in Bangladesh is needed to explore whether improved service utilization in home-based settings translates into sustained improvements in child growth and development metrics.”

*4. Addressing Potential Biases
• The study excludes urban areas, which limits generalizability. Although this is mentioned as a limitation, a comparison with urban settings from prior research could strengthen the discussion*

Our response: Edited as suggested, read as follows [lines 623-627]:

“Firstly, it was conducted in only a few subdistricts of rural Bangladesh, excluding urban areas, potentially compromising the representation of the country's overall GMP utilization scenario, similar to other prior research studies from LMICs [13]. However, two recent studies conducted in South Asia and Africa showed no urban-rural differences in GMP utilization status [25, 30].”

*• Selection bias due to the exclusion of children with severe malnutrition could affect findings Further discussion on this would improve credibility*

Our response: Edited as suggested, reads as follows [lines 510-514]:

“Our study included only apparently healthy, disease-free children, which might introduce a selection bias and limit the generalizability of the findings to children with underlying health conditions, including severe malnutrition. We did not collect information from excluded children and thus could not assess the impact on study findings and the GMP service utilization matrix.”

*5. Visual Presentation of Data
• Figures and Tables:
o The flow diagram (Figure 1) could be more visually engaging*.

Our response: Edited as suggested, reads as follows:

*o Tables 3-5 should be condensed where possible to improve readability.*

Our response: Edited as suggested and aligned with reviewer SV’s edits, reads as follows:

**Table 3.** **Levels of GMP service utilization among individuals in facility (**N = 1,519) **and home-based (**N = 1,519) **GMP programs (**N = 1,519)

| **Indicators** | **Home-based GMP N (%)** | **Facility-based GMP N (%)** | **RR (95% CI)** | **p-value** |
| --- | --- | --- | --- | --- |
| Caregivers received a GMP card | 926 (60.9) | 0 (0) | - | - |
| The child was weighed at least once | 1231 (81.0) | 311 (20.5) | 4.0 (3.6, 4.4) | <0.001 |
| The child length was measured at least once | 51 (3.36) | 09 (0.59) | 5.7 (2.8, 11.5) | <0.001 |
| Caregivers got child growth monitoring-specific counseling | 837 (55.2) | 88 (5.4) | 9.5 (7.7, 11.7) | <0.001 |
| The child received all major components of GMP (length, weight, and growth monitoring-specific counseling) altogether | 30 (1.97) | 0 (0) | - | - |
| Caregivers utilized GMP services | 603 (39.7) | 0 (0) | - | - |

Growth monitoring and promotion, GMP; confidence interval, CI; Risk Ratio, RR.

**Table 4:** **Barriers to GMP service utilization among individuals in the facility (**N = 1,519) **and home-based (**N = 1,519) **GMP^a^ programs**

| **Indicators** | **Home-based GMP N (%)** | **Facility-based GMP N (%)** | **RR (95% CI)** | **p-value** |
| --- | --- | --- | --- | --- |
| The caregiver was preoccupied with household chores and did not go for the GMP service | 56 (3.7) | 5 (0.3) | 11.2 (4.5, 27.9) | <0.001 |
| The health facility was closed/no healthcare provider at the facility | 14 (0.9) | 3 (0.2) | 4.6 (1.3, 16.2) | 0.015 |
| Caregivers' lack of interest in GMP services at an individual level | 668 (44.0) | 834 (54.9) | 0.7 (0.6, 0.9) | 0.006 |
| Lack of knowledge of GMP service availability at the nearest health facility | 798 (52.5) | 919 (60.5) | 0.9 (0.8, 0.9) | <0.001 |

Growth monitoring and promotion, GMP; confidence interval, CI; Risk Ratio, RR.

^a^Multiple responses by caregivers.

**Table 5. Predictors of GMP service utilization among caregivers at the home-based GMP programs**

| **Characteristic^a^** | **Characteristic category** | **RR (95% CI)** | **p-value** | **ARR (95% CI)** | **p-value** |
| --- | --- | --- | --- | --- | --- |
| Caregiver having heard about GMP or GMP card | No^1^ | - | - | - | - |
|  | Yes | 42.4 (20.3, 88.6) | <0.001 | 37.4 (17.8, 78.5) | <0.001 |
| Caregiver being a member of an association/NGO/health program | No^1^ | - | - | - | - |
|  | Yes | 2.6 (2.1, 3.2) | <0.001 | 1.3 (1.1, 1.5) | 0.001 |
| Caregiver's low interest in GMP services | No^1^ | - | - | - | - |
|  | Yes | 0.5 (0.4, 0.7) | <0.001 | 0.7 (0.5, 0.9) | 0.001 |

RR: Risk ratio, CI: Confidence interval, ARR: Adjusted risk ratio, Growth monitoring and promotion, GMP; Non-governmental organization, NGO; and 1 = reference category

^a^The Model included caregivers who heard about GMP or GMP cards, were members of an NGO, and lacked interest in GMP service.

*o A graphical summary of key results (e.g., a bar chart showing GMP utilization disparities) would enhance clarity.*

Our response: Added as suggested (line 401), and uploaded as Fig 2:

|  | Home-based GMP N (%), N = 1,519 | Facility-based GMP N (%), N = 1,519 |
| --- | --- | --- |
| Caregivers heard about GMP or GMP card | 66.8 | 0.4 |
| Caregivers received a GMP card | 60.9 | 0 |
| Caregivers got child growth monitoring-specific counseling | 55.2 | 5.4 |
| Caregivers utilized GMP services | 39.7 | 0 |

**Fig 2. Caregivers’ knowledge and level of GMP service utilization**

*6. Policy & Implementation Implications*

*• The study suggests NGO-driven GMP services improve utilization, but how can this be implemented at scale?*

Our response: Edited as suggested (lines 594-601):

“Integrating NGO-led initiatives into the public health system could enhance the continuity and reach of services. This could involve joint training of health workers, shared protocols, and coordinated referral systems between home- and facility-based services. The success of NGO-supported, home-based GMP highlights the potential of community-based approaches, but scaling these models requires embedding them within national health systems. A hybrid approach, combining NGO innovations with government structures, could be effective, with shared workforce development and unified monitoring tools. ”

*• Policy recommendations should be expanded:
o Should the government adopt a hybrid approach with NGO support?*

Our response: Edited as suggested (lines 599-601):

“A hybrid approach, combining NGO innovations with government structures, could be effective, with shared workforce development and unified monitoring tools.”

*o What cost-effectiveness considerations exist for scaling up home-based GMP?*

Our response: Edited as suggested (lines 601-606):

“To assess the feasibility of this hybrid government-NGO model, cost-effectiveness analyses should compare home-based and facility-based GMP delivery costs and outcomes. Evidence showed home-based GMP can become cost-effective when integrated with existing health platforms [14, 39, 40]. Though start-up costs may be high, integration with existing services and use of digital tools can improve cost-effectiveness and scalability over time [40].”

**Responses to the comments made by Reviewer #6:**

*While the methods presented are very detailed, I have some concerns about whether the authors of this paper are trying to report too much in a single study for example I am not sure as to why there was a need to report mean birth weight and birth length in this study as it did not directly lend to the objectives of 1st determining whether there was a gap and 2. establishing the barriers. I suggest only reporting data that directly answers these two objectives.*

Our response: Thank you for your query and suggestion. We did not measure and report birth weight and birth length of the child. We reported child weight and length at enrolment to understand and compare any difference in anthropometric incidence of the children from home and facility-based GMP area (lines 357-360), the comparison was done as per suggestion from 1^st^ round reviewer’s comment:

“Children's length-for-age Z-score (LAZ) and weight-for-age Z score (WAZ) was comparable among the groups. Children from home-based GMP area had significantly lower weight-for-length/height Z-score (WHZ) compared to facility-based area children (mean WHZ: -0.48 versus -0.4, p=0.04).”

*Further than knowledge, I am not clear what other barriers were explored and how they were explored.*

Our response: Our apologies for this confusion. Edited as suggested (lines 199-202):

“The caregivers’ barriers to access and utilization of GMP services were explored using a semi-structured survey questionnaire and during qualitative interviews. The survey questionnaire included multiple response options.”

*There was mention of thematic analysis but there is no reporting of the data and outcomes of the thematic analysis using accepted guidelines.*

Our response: Edited as suggested (lines 287-297, 322-340):

“The theoretical framework guiding this study is the Health Belief Model (HBM), which emphasizes individuals’ perceptions of health risks, the benefits of preventive actions, and the barriers to adopting these actions. In the context of this study, the HBM helped to understand caregivers' motivations and decision-making regarding GMP services. It informed the development of research questions related to perceived severity, susceptibility, benefits, and barriers to GMP service utilization. This framework also guided the identification of key themes during the qualitative analysis, focusing on the barriers caregivers face and the factors that influence their engagement with GMP services. By using the HBM, the study aimed to provide a structured understanding of caregivers' behaviors and attitudes toward health service utilization in rural Bangladesh.”

“Qualitative analysis involved thematic descriptions, analysis, and respondent quotations. Transcribed interviews were coded based on predetermined themes (a priori), subthemes, and emerging issues. The coding process began with thorough transcription and review of the data to familiarize them with the content. A preliminary coding scheme was developed from emerging patterns and concepts. These codes were manually applied to the transcripts, with similar codes grouped into categories. Themes were derived through an iterative process of constant comparison, refining and redefining codes as the analysis progressed. The final themes were shaped by both the research questions and the data. Team discussions and member checks were used to validate the consistency and relevance of the themes. Trustworthiness was ensured through four principles: credibility, transferability, conformability, and dependability [24]. To ensure credibility, prolonged engagement and member checks were used, along with data triangulation to confirm findings. Transferability was enhanced by providing detailed descriptions of the research context, participants, and methods, allowing for comparison to similar settings. Conformability was maintained through an audit trail and peer debriefing to ensure findings were grounded in the data. Dependability was ensured by thoroughly documenting the research process, allowing for replication and consistency over time. Intercoder or synchronic reliability was utilized to gauge the agreement between two independent coders of the data. An agreement was assessed during the analysis when the researchers independently coded the same interviews. There were no discrepancies observed among the coders.”

**Responses to the comments made by Reviewer #7:**

1. *The authors have sufficiently addressed the feedback from the previous review round, and I believe this manuscript is now suitable for publication.*

Our Response: Thank you for your comments.

*2. The manuscript presents a technically solid piece of scientific research; however, the conclusions chapter requires expansion.*

Our Response: Edited as suggested, read as follows (lines 638-655):

“This study provides important insights into the utilization dynamics of Growth Monitoring and Promotion (GMP) services in rural Bangladesh, highlighting distinct disparities between home-based and facility-based service delivery models. While home-based services showed relatively better utilization and caregiver knowledge, overall engagement remained suboptimal, pointing to deeper structural, behavioral, and systemic challenges. The complete absence of utilization in facility-based settings underscores the urgent need for strategic reforms that address supply chain issues, caregiver perceptions, service accessibility, and trust in service providers.

Our findings emphasize that improving GMP uptake requires more than availability—it necessitates the thoughtful integration of community-based strategies, caregiver education, consistent health worker engagement, and system-level coordination between NGOs and government services. While the study is limited by its cross-sectional design, rural-only focus, and short duration, it contributes novel evidence on GMP service delivery gaps and caregiver preferences in Bangladesh. Future research should build on these findings through longitudinal and intervention-based studies to identify sustainable approaches for improving GMP coverage and impact, particularly among underserved populations. Addressing sociocultural norms, strengthening facility-based systems, and aligning services with caregiver motivations will be critical to ensuring that GMP fulfills its preventive health potential.”

*3.The statistical analysis has been conducted appropriately and rigorously.*

Our Response: Thank you for your comments.

*4.The authors have made all the data supporting their manuscript's findings fully accessible.*

Our Response: Thank you for your comments.

*5.The manuscript is presented clearly and is written in standard English.*

Our Response: Thank you for your comments.

**Responses to the comments made by Tamir Velner**

1. *I suggest including the word "Barriers" among the keywords in the abstract.*

Our Response: We added “Barrier” among the keywords in the abstract (line 57).

2. *The number of sources cited throughout the article is insufficient. I recommend adding more current sources throughout the various sections of the article.*

Our Response: We added more recent references relevant to the topic of discussion throughout the various sections of the article published last year:

25. Upadhyay JP, Paneru DP, Sharma YP, Khadka NR. Utilization and associated factors of growth monitoring and promotion services among young children in Gorkha district of Nepal. BMC Nutrition. 2024;10(1):164. doi: 10.1186/s40795-024-00978-z.

30. Edae BS, Yazew T, Disasa HD, Kuyu CG. Growth Monitoring and Promotion Service Utilization and Associated Factors Among Mothers/Caregivers of Children 0-23 Months in Sude District, Southeast Ethiopia. Matern Child Nutr. 2025;21(2):e13769. Epub 20241127. doi: 10.1111/mcn.13769. PubMed PMID: 39604193; PubMed Central PMCID: PMCPMC11956038.

35. Sarker T, Ahmed S, Rahman S, Chakraborty B. Maternal misperception of under-five children weight status and associated factors: A cross-sectional study. Matern Child Nutr. 2024:e13674. Epub 20240528. doi: 10.1111/mcn.13674. PubMed PMID: 38804268.

39. Rockers PC, Leppänen JM, Tarullo A, Coetzee L, Fink G, Hamer DH, et al. Evaluation of a community health worker home visit intervention to improve child development in South Africa: A cluster-randomized controlled trial. PLoS Med. 2023;20(4):e1004222. Epub 20230414. doi: 10.1371/journal.pmed.1004222. PubMed PMID: 37058529; PubMed Central PMCID: PMCPMC10146459.

40. Liang Y, Peng X, Sun MA. Long-Term Impacts of Growth and Development Monitoring: Evidence from Routine Health Examinations in Early Childhood2024 12 March 2025.

*3. There are instances where the same sources are repeated within the same paragraph and occasionally in other parts of the article. I suggest significantly diversifying the sources used.*

Our Response: We added additional references where available and tried to diversify sources based on the availability and relevancy of resources.

*4. It's crucial to include up-to-date references from studies published within the last years.*

Our Response: Responded in comment #2.

*5. Has the semi-structured questionnaire, which was pretested, been validated by content experts? Was the measurement instrument developed based on scientific literature? Has it undergone translation from Bangladeshi to English and then back to Bangladeshi?*

Our Response: Edited as suggested (lines 215-221), read as follows:

“The semi-structured questionnaire was developed based on a review of relevant scientific literature and existing tools used in similar low- and middle-income country contexts. Its content validity was reviewed by a panel of nutrition and public health experts in Bangladesh. The original tool was prepared in English, translated into Bangla, and then back-translated to English by an independent translator to ensure accuracy and consistency. It was pretested in a comparable setting to assess clarity, cultural appropriateness, and flow, and subsequently refined before final use.”

*6. Are researchers completely confident that no other study has ever reported a lack of GMP use in facilities—a situation that is unique to this setting and contrasts with findings from existing LMIC GMP programs? I recommend rephrasing this sentence in a more modest and careful manner.*

Our Response: Edited as suggested, read as follows (line 522-524):

“Few studies have documented such findings, which is unique to this setting and contrast with the existing LMIC GMP program findings [9, 28, 29].

*7. The conclusions section is limited to just one paragraph, making it excessively concise and in need of substantial expansion. Furthermore, it offers only one sentence of recommendations for policymakers focused on shaping public health strategies. It points out that simply prioritizing among the nation's decision-makers for the integration of home-based GMP services is insufficient.*

Our Response: Edited as suggested, read as follows (lines 638-655):

“This study provides important insights into the utilization dynamics of Growth Monitoring and Promotion (GMP) services in rural Bangladesh, highlighting distinct disparities between home-based and facility-based service delivery models. While home-based services showed relatively better utilization and caregiver knowledge, overall engagement remained suboptimal, pointing to deeper structural, behavioral, and systemic challenges. The complete absence of utilization in facility-based settings underscores the urgent need for strategic reforms that address supply chain issues, caregiver perceptions, service accessibility, and trust in service providers.

Our findings emphasize that improving GMP uptake requires more than availability—it necessitates the thoughtful integration of community-based strategies, caregiver education, consistent health worker engagement, and system-level coordination between NGOs and government services. While the study is limited by its cross-sectional design, rural-only focus, and short duration, it contributes novel evidence on GMP service delivery gaps and caregiver preferences in Bangladesh. Future research should build on these findings through longitudinal and intervention-based studies to identify sustainable approaches for improving GMP coverage and impact, particularly among underserved populations. Addressing sociocultural norms, strengthening facility-based systems, and aligning services with caregiver motivations will be critical to ensuring that GMP fulfills its preventive health potential.”

We hope that in its present form the manuscript is acceptable for publication in PLOS ONE. The material has not and will not be offered elsewhere for possible publication, as long as it is under PLOS ONE journal consideration.

Sincerely,

Dr. Muttaquina Hossain

Associate Scientist

Nutrition Research Division, icddr,b

Email: [muttaquina@icddrb.org](mailto:muttaquina@icddrb.org); [muttaquina@gmail.com](mailto:muttaquina@gmail.com)

Mobile: +88-01923758956
