# Supplementary material for: Growth monitoring and promotion program services utilization patterns between home-based and facility-based delivery methods: A comparative analysis
Source: PLoS One. 2025 Jun 5;20(6):e0324918. doi: 10.1371/journal.pone.0324918 (PMC12140421; doi:10.1371/journal.pone.0324918)
Supplement: Attachment — Submitted filename: Response to the 3rd round review comments.docx [file pone.0324918.s013.docx]

30 April 2025

Zeheng Wang
Academic Editor
PLOS ONE

California, USA

RE: PONE-D-24-20319R2: Growth monitoring and promotion program services utilization patterns between home-based and facility-based delivery methods: a comparative analysis

Dear Academic Editor,

We thank you for your response to our manuscript. Please find attached a revised version, in response to your invitation to resubmit the paper to PLOS ONE if we felt that we could satisfactorily address the concerns of the reviewers. We have substantially modified the manuscript, considering the comments from the four reviewers.

Our response to the comments is presented using the following structure: The original editorial / referee comment is copied into this letter in italics. Our response to the comment is written below in normal text format. In response to your invitation to resubmit, we have substantially modified the manuscript, considering the comments from the Academic Editor and one reviewer. We have removed the reference of the retracted paper and replaced with a new reference, and revised the reference list, as per Academic Editors suggestion. In the case of reviewer 8, we addressed her comments inside the manuscript and copied them in this rebuttal letter. In case a revision to the manuscript text has been made, we give the appropriate page number(s). Changes in the text have been indicated using yellow text highlighting in the manuscript and copied into this letter in the same format. The highlighted text file has been uploaded into your electronic system as a “Revised Manuscript with Track Changes”.

**Responses to the comments made by Academic Editor:**

*Please review your reference list to ensure that it is complete and correct. If you have cited papers that have been retracted, please include the rationale for doing so in the manuscript text, or remove these references and replace them with relevant current references. Any changes to the reference list should be mentioned in the rebuttal letter that accompanies your revised manuscript. If you need to cite a retracted article, indicate the article’s retracted status in the References list and also include a citation and full reference for the retraction notice.*

Our response: Thank you for this important observation. We have reviewed our reference list thoroughly. In our initial submission, we cited the 2000 Cochrane Review by Panpanich and Garner on growth monitoring. However, we acknowledge that this review has been withdrawn and superseded by an updated Cochrane Review.

Accordingly, we have removed the citation of the withdrawn review and have replaced it with the updated and current Cochrane Review. This change is also reflected in the revised manuscript text and reference list and read as follows (lines 10, 26, 29, 40, 83, 127, 135, 535, 583):

“1. Taylor M, Tapkigen J, Ali I, Liu Q, Long Q, Nabwera H. The impact of growth monitoring and promotion on health indicators in children under five years of age in low- and middle-income countries. Cochrane Database Syst Rev. 2023;10(10):Cd014785. Epub 20231012. doi: 10.1002/14651858.CD014785.pub2. PubMed PMID: 37823471; PubMed Central PMCID: PMCPMC10568659.”

**Responses to the comments made by reviewer 8:**

*In the uploaded manuscript, I have included three minor comments on your Discussion section and highlighted their text in light blue so that they can be detected easily.
Also: Well done dear authors. My initial assessment was that your study brings value to the healthcare services field needs serious attention to stronger scientific writing and organization. Now, after rounds of review and your hard work, its writing matches its value. Thank you for following my numerous recommendations.*

Our response: We appreciate your kind observation. We now have revised the discussion section as suggested.

*Please dedicate the entire first paragraph of the discussion section to: 1) briefly restating study aims 2) briefly restating main study findings. Including one sentence on the study's importance and reserach gap is appreciated, which you have already included.*

*Don't include any interpretations or comparisons with the literature here, save them for the following pargaraphs.*

*In the following pargaraphs, each main fdining is brief restated and comapred to the literature and explained in that context, as you have already mostly done so.*

*So please just revise this frist paragraph to include these 3 features: 1) briefly pointing out the study's importance and reserach gap 2) briefly restating study aims 3) briefly restating main study findings*

Our response: Edited as suggested, reads as follows (lines 423-430):

“This study assessed caregivers’ knowledge, perceptions, utilization, and barriers related to growth monitoring and promotion (GMP) services in rural Bangladesh. It compared GMP services delivered at home with those delivered at health facilities. The study addresses a key evidence gap by providing one of the first comparative analyses of these two service delivery platforms. Findings showed that caregivers in home-based GMP areas had greater awareness of GMP, received more GMP cards, and were more likely to have their children measured and counseled. Overall GMP service utilization was 40% in home-based areas, compared to 0% in facility-based areas. This finding underscores a stark contrast in service reach and engagement.”

*This is a paragraph on your study's limitations and the ways you strived to mitigate them. Such paragraphs are typically included at the end of the discussion section. You aready have another one of these paragraphs at the end of your discussion, so I suggest you move this one to the end as well and merge the to paragraphs together. Also please make them more concise after merging them.*

Our response: Edited as suggested, reads as follows (lines 517-535):

“The validity and reliability of the study findings may have been affected by several factors. Only one NGO provided home-based GMP services, and its longstanding presence in the community could have influenced caregivers’ service uptake. However, similar sociodemographic characteristics among participants have likely reduced this bias. Despite the NGO’s role and the accessibility of home-based services, utilization remained low, suggesting limited influence. We exclude children with chronic illness, severe malnutrition, and twins, which may introduce selection bias and limit generalizability. No data were collected on excluded children, so their impact on findings and GMP utilization patterns could not be assessed. Twins were excluded to minimize information bias. Though the study period was short, it still offers important insights into GMP utilization in rural settings. The study was conducted in a few rural subdistricts, excluding urban areas, which may limit the representativeness of national GMP patterns. However, two recent studies from South Asia and Africa reported no urban–rural differences in GMP utilization [25, 28]. The cross-sectional design limits causal inference between caregivers’ utilization and associated factors. The absence of longitudinal follow-up also prevents understanding of longer-term impacts. Facility-based GMP services were not utilized at all, preventing analysis of predictors in that group. Differences in baseline characteristics between groups may have contributed to outcome differences despite statistical adjustments. Thus, results should be interpreted with caution. Still, the study contributes novel evidence from a context where comparable government or research data are lacking [1].”

*I belive you should move this paragraph so that it comes before the "future research"one that is currently located above it.*

Our response: Edited as suggested, reads as follows (lines 536-550):

“Future research should explore these factors further to provide a more comprehensive understanding of GMP utilization dynamics. In particular, studies should explore sociocultural and behavioral influences on caregivers’ decisions—such as preferences for curative over preventive care and trust in various service providers. Further investigation is also needed into the effectiveness of health worker communication strategies, the role of incentives, and the influence of service accessibility factors like distance, cost, and competing time demands. Comparative research across urban and rural contexts would also be valuable for identifying context-specific barriers and informing the design of more tailored, equitable GMP interventions. While this study did not assess long-term outcomes, prior evidence from LMICs suggests that consistent GMP engagement—particularly when combined with responsive counseling—can lead to earlier identification of growth faltering, improved caregiver feeding practices, and ultimately better child nutrition, development outcomes and productivity [11, 28, 39]. Longitudinal research in Bangladesh is needed to explore whether improved service utilization in home-based settings translates into sustained improvements in child growth and development metrics.”

We hope that in its present form the manuscript is acceptable for publication in PLOS ONE. The material has not and will not be offered elsewhere for possible publication, as long as it is under PLOS ONE journal consideration.

Sincerely,

Dr. Muttaquina Hossain

Associate Scientist

Nutrition Research Division, icddr,b

Email: [muttaquina@icddrb.org](mailto:muttaquina@icddrb.org); [muttaquina@gmail.com](mailto:muttaquina@gmail.com)

Mobile: +88-01923758956
